# Antennal Proteome of the *Solenopsis invicta* (Hymenoptera: Formicidae): Caste Differences in Olfactory Receptors and Chemosensory Support Proteins

**DOI:** 10.1093/jisesa/ieaa118

**Published:** 2020-10-24

**Authors:** Jaee Shailesh Shah, Robert Renthal

**Affiliations:** Department of Biology, University of Texas at San Antonio, San Antonio, TX

**Keywords:** Solenopsis, proteomics, olfactory receptors, odorant degradation, male caste evolution

## Abstract

Little is known about the expression pattern of odorant and pheromone transporters, receptors, and deactivation enzymes in the antennae of ants carrying out different tasks. In order to begin filling in this information gap, we compared the proteomes of the antennae of workers and males of the red fire ant, *Solenopsis invicta* Buren (Hymenoptera: Formicidae). Male ants do not perform any colony work, and their only activity is to leave the nest on a mating flight. Previous studies showed that male ants express fewer types of odorant receptors than workers. Thus, we expected to find large differences between male and worker antennae for expression of receptors, transporters, and deactivators of signaling chemicals. We found that the abundance of receptors was consistent with the expected caste-specific signaling complexity, but the numbers of different antenna-specific transporters and deactivating enzymes in males and workers were similar. It is possible that some of these proteins have antenna-specific functions that are unrelated to chemosensory reception. Alternatively, the similar complexity could be a vestige of ant progenitors that had more behaviorally active males. As the reduced behavior of male ants evolved, the selection process may have favored a complex repertoire of transporters and deactivating enzymes alongside a limited repertoire of odorant receptors.

## Graphical Abstract

**Graphical Abstract:**
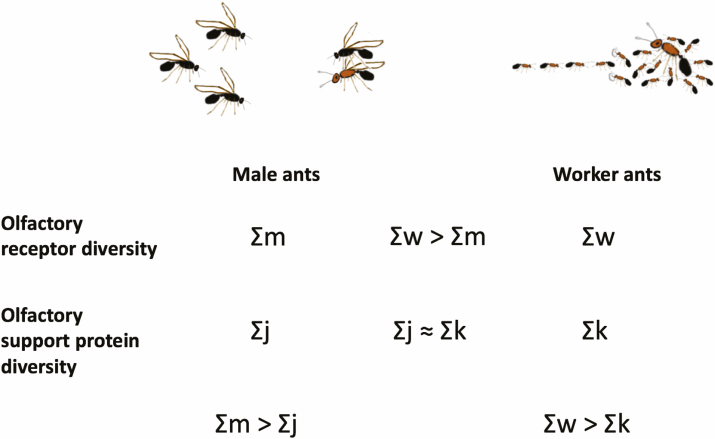
The diversity of olfactory support proteins is about the same in worker and male fire ants, in contrast to the diversity of olfactory receptors, which is much higher in workers.

Social behavior in ant colonies depends, in part, on chemical signaling between individual colony members. The complexity of these interactions is mirrored by ant genomes, which code for large numbers of odorant receptors (ORs). For example, recent analyses (McKenzie et al. 2016, Cohanim et al. 2018) identified about 470 different OR sequences in the genome of the red fire ant, *Solenopsis invicta* Buren. The physiological process of chemical signal reception may also require, in the case of substances with low water-solubility, various hydrophobic ligand-binding proteins, such as odorant-binding proteins (OBPs) and chemosensory proteins (CSPs). Some of these proteins have been proposed as transporters of signaling molecules to the ORs (Pelosi et al. 2014). In the *S. invicta* genome, 24 OBPs (Gotzek et al. 2011, Pracana et al. 2017) and 21 CSPs (Kulmuni et al. 2013) have been identified. In addition to transporters and receptors, insect chemosensory reception requires enzymes that deactivate the semiochemicals (Kaissling 2009, Kaissling 2013). These enzymes have not often been studied at the genomic level in social insects, but there are 100 or more sequences in some insect genomes, of which an unknown number are involved in signaling molecule deactivation (Feyereisen 2012, Younus et al. 2014). Little is known about the expression pattern of transporters, receptors, and deactivation enzymes in the antennae of ants carrying out different tasks, such as foraging, brood care, and reproduction. In order to begin filling in this information gap, we analyzed the proteomes of the antennae of *S. invicta* workers and males. Male ants do not perform any colony work, and their only activity is to leave the nest on a mating flight, after which they die. Previous studies showed that male ants are tuned to a narrower range of chemicals than female ants. For example, male ant antennae have fewer types of sensillae than workers (Renthal et al. 2003, McKenzie et al. 2016). Also, male ant antennae express fewer ORs than workers and their brain antennal lobes have fewer glomeruli (Zube and Rossler 2008, Nakanishi et al. 2010, McKenzie et al. 2016). Thus, we expected to find large differences between male and worker antennae for expression of receptors, transporters, and deactivators of signaling chemicals. As described in this paper, we found that the abundance of receptors showed the expected correlation with caste-specific signaling complexity, but the numbers of different antenna-specific transporters and deactivating enzymes in males and workers were similar. This similar complexity may be a vestige of ant progenitors that had more behaviorally active males. As the reduced behavior of male ants evolved, the selection process appears to have favored a complex repertoire of transporters and deactivating enzymes alongside a limited repertoire of ORs.

## Materials and Methods

### Collection of Ant Colonies

Polygyne *S. invicta* colonies were collected in Bexar County, Texas, using the floatation method (Jouvenaz et al. 1977), and maintained in a controlled environment in plastic trays containing Petri dish nests. The ant colonies were fed with water, 10 % sucrose solution, and beef liver.

### Dissection and Protein Extraction

Prior to dissection, the ants were anesthetized with CO_2_ and then cooled to 4°C for ~ 1 hr. For sodium docecyl sulfate polyacrylamide gel electrophoresis (SDS–PAGE), antennae from workers (50 workers, 100 antennae), males (115 males, 230 antennae) and the tibiae from worker legs (13 workers, 78 tibiae) were dissected on a −20°C cold plate, and the appendages were held at 0°C until the extraction step. The proteins were extracted from pooled worker or male antennae or pooled worker tibia by grinding the tissue at 23°C in ~ 60 µl of 3% SDS solution using a 3 cm diameter ceramic mortar and a small ceramic pestle. The ant proteins were separated from insoluble material by centrifugation in a Sorvall MC 12V minicentrifuge at 10,000 rpm for 5 min. This extraction method probably extracts relatively more water-soluble and membrane proteins and fewer fibrous and cuticular proteins. One biological replicate was analyzed from each pool.

For blue native polyacrylamide gel electrophoresis analysis (BN-PAGE), the antennae of workers (103 workers, 206 antennae; or 200 workers, 400 antennae) and males (67 males, 134 antennae) were extracted by grinding at 23°C in ~80 µl of extraction solution consisting of 78 mM dodecyl maltoside, 49.8 mM Bis-tris,16 mM HCl and 14% of 1X complete Mini protease inhibitor cocktail (Millipore Sigma, St. Louis, MO). The extract was centrifuged at 10,000 rpm for 5 min. In one experiment, the extracted proteins were concentrated using a 100 kDa Amicon Ultra centrifugal ultrafiltration unit (Millipore Sigma) at 14,000 rpm for 5 min to a volume of 13 µl. Two biological replicates of pooled worker antennae were analyzed, and one biological replicate of pooled male antennae was analyzed.

### Electrophoresis

SDS–PAGE of the extracted proteins was performed using NuPAGE 4–12% acrylamide Bis-tris gels (ThermoFisher, Carlsbad, CA). BN-PAGE of the extracted proteins was performed using NativePAGE Novex 4–16% Bis-Tris gels (ThermoFisher). A native PAGE unstained protein standard (ThermoFisher) was used as soluble protein standard. A membrane protein standard was prepared by following the protocol of Wittig et al. (Wittig et al. 2010).

### In-Gel Trypsin Digestion

For the subsequent mass spectrometry analysis, proteins were digested by trypsin using an in-gel protocol (Renthal et al. 2017). The SDS–PAGE gels, which had been run approximately 4 cm, were divided into four roughly equal segments. For the BN-PAGE gels, segments were cut to collect Orco-OR complexes in the molecular weight range expected for tetramers to octamers. In order to select regions of the native gels that would contain olfactory receptor tetramers, we calculated the average OR molecular weight as 44 kD for 111 sequences of *S. invicta* odor-specific olfactory receptors listed in NCBI above 35 kD (assuming that sequences of ORs with molecular weights less than 35 kD may be incomplete).

### Mass Spectrometry

Tryptic peptides obtained from in-gel trypsin digestion of antennal and tibial protein extracts were analyzed by a nano-reverse phase liquid chromatography (LC) electrospray ionization tandem mass spectrometry (MS) system, consisting of an UltiMate 3000 Nano LC system and an LTQ- Orbitrap Elite mass spectrometer (ThermoFisher, San Jose, CA), using instrument settings similar to those described previously (Renthal et al. 2017). The mass spectra of the peptide sequences were compared with the protein sequences of *S. invicta* in the National Center for Biotechnology Information (NCBI) protein database (2018 data) by using Mascot software (version 2.6; Matrix Science, Boston, MA). Protein hits were counted if the Mascot ions scores were above the threshold of statistical significance for identity (*P* < 0.05) (Perkins et al. 1999). The peptide false discovery rate was 1%, determined using the same database with reversed sequences. For protein hits carrying headers in the NCBI protein database such as ‘uncharacterized’ or ‘unknown’, the ‘Features’ section of the NCBI data file was examined, and often annotations that identified the proteins were found. In the absence of further annotation, uncharacterized sequences were subjected to BLAST searches against Arthropoda, and also Swiss-Model was used for three-dimensional homology searches. In many cases, these searches yielded tentative identifications of uncharacterized or unknown sequences.

Label-free quantification was done using the exponentially multiplied protein abundance index (emPAI). For secreted proteins (identified by SignalP software, cbs.dtu.dk) emPAI was calculated by the method of Ishihama et al. (Ishihama et al. 2005) as previously described (Renthal et al. 2017). For cytoplasmic proteins, emPAI was calculated by Mascot software. The emPAI values were normalized to glyceraldehyde 3-phosphate dehydrogenase (GAPDH). In order to account for variability in the volumes of samples extracted from gel slices that were analyzed on the LTQ-Orbitrap Elite, the emPAI values were corrected for variations in the amount of trypsin autolysis products detected by mass spectrometry in each sample, assumed to be inversely proportional to relative sample volume. For each gel slice analyzed, emPAI was calculated for porcine trypsin. In addition, the relative amount of trypsin was calculated using the Top3 method of Silva et al. (Silva et al. 2006), in which the intensities of the three highest intensity ions are averaged. These intensities were obtained by converting the Thermo RAW files to mgf format, using MSconvert software (ProteoWizard), and then collecting the precursor ion intensities of the trypsin autolysis MS^2^ ions using custom software. The variation in trypsin between samples, measured by averaging the Top3 and emPAI values, was taken as a ratio to the section containing GAPDH, and this was used to correct the GAPDH-normalized emPAI values of each protein. The average sample volume error factor, calculated by this method, was 1.2 ± 0.4.

Cytochrome P450 sequences that were identified in *S. invicta* antennae and tibiae were compared with all *S. invicta* P450 sequences found in the NCBI database and with a published list of *Nasonia vitripennis* P450s (Oakeshott et al. 2010) by generating a maximum likelihood phylogenetic tree from ClustalO-aligned sequences using RAxML software (Stamatakis 2014) run on CIPRES 3.1. The tree output was displayed in CLC Sequence Viewer 8 software (Qiagen).

A gene ontology (GO) analysis was done using g:Profiler software (Raudvere et al. 2019), with a significance threshold of 0.05.

## Results

### Antennal and Tibial Proteomes

The worker antennal proteome was compared with the proteomes of male antennae and worker tibiae. We assume that proteins common to all three appendages are unlikely to be chemoreception-specific. Tibia are known to have chemosensory sensilla (Mitchell et al. 1999, Newland et al. 2000). However, the chemosensory functions of the tibia are probably limited to gustatory activity. Consistent with this, in *S. invicta* tibiae we could not detect any odorant receptor coreceptor (Orco) or sensory neuron membrane protein (SNMP), in contrast to antennae (see below). Proteins such as OBPs or P450s belong to families having some members with chemoreception-related functions and others with non-chemoreception functions. When members of these families were detected in both antennae and tibiae, we considered them less likely to be involved in chemoreception than proteins detected exclusively in the antennae.

**Fig. 1.**
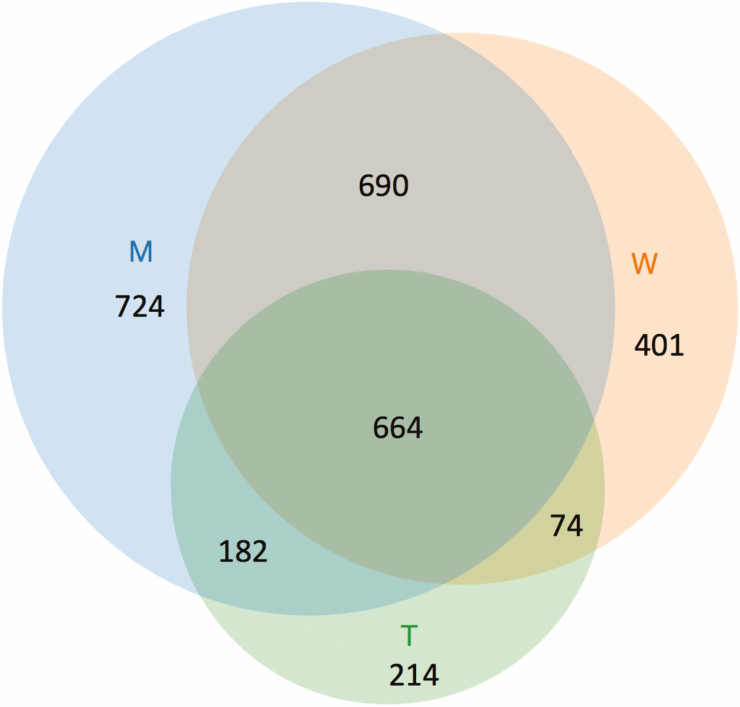
Venn diagram of identified proteins in *S. invicta* worker antenna (W), male antenna (M) and worker tibia (T).

A total of 2,949 proteins were identified by Mascot software as statistically significant (*P* < 0.05). Of these, 664 were found in both male and worker antennae and worker tibiae, and 690 were found in both worker and male antennae but not in tibiae. In addition, 74 proteins were in both worker antennae and tibiae, and 182 proteins were in both male antennae and worker tibiae. Worker antennae had 401 proteins not found in male antennae or worker tibiae, and male antennae had 724 proteins not found in worker antennae or worker tibiae. Worker tibiae had 214 proteins not found in worker or male antennae. A Venn diagram is shown in Fig. 1, and a list of the most abundant proteins identified is shown in Supp Table S1 (online only). Among the most abundant antennal proteins are hydrophobic ligand-binding proteins and hydrophobic ligand deactivation enzymes, which are likely to be involved in chemosensation, as discussed below.

Through GO analysis, the proteins identified in male antennae, worker antennae, and worker tibia, were associated with 202 GO terms for biological processes, of which 35 were in worker and male antennae but not in worker tibiae, 7 in worker tibiae but not male or worker antennae, 41 only in male antennae, 5 only in worker antennae, 10 in male antennae and worker tibiae but not in worker antennae, and 2 in worker antennae and worker tibiae but not in male antennae (Supp Table 2 [online only]). The largest group of over-represented GO terms in antennae compared with tibiae are related to actin or other fiber polymerization and/or depolymerization: 21 were GO terms in male antennae and 6 were GO terms in worker antennae, but none were in tibiae.

### Olfaction-Related Hydrophobic Ligand-Binding/Transport Proteins

#### Odorant-Binding Proteins

Mass spectra matching 11 of the 24 known *S. invicta* OBPs (Gotzek et al. 2011, Pracana et al. 2017) were detected in antennae and tibiae (Table 1). Ten were detected in worker antennae. Six of the same proteins were detected in the male antennae, and four were detected in the tibiae. One additional OBP was detected only in the tibiae. The four tibial OBPs that were also found in antennae probably are not involved in chemoreception or are multi-functional proteins. The six worker and four male OBPs that are exclusively antennal potentially could be involved in odorant reception. Three of the OBPs detected in the antennae are expressed from genes on the social chromosome (Pracana et al. 2017). However, all three were also detected in tibiae, so they probably are not involved in olfaction or are multifunctional.

**Table 1.** Odorant-binding proteins

NCBI accession number	Name^*a*^	Worker antenna^*b*^	Male antenna^*b*^	Worker tibia^*b*^
XP_011157711.1	OBP3^*c,d*^	73	6.5	640
ADX94399.1	OBP2	1.9	1.4	0
ADX94401.1	OBP5	0.95	0.25	0
ADX94412.1	OBP16^*d*^	0.45	0	0.39
ACI30688.1	OBP1	0.45	0.38	0
ADX94403.1	OBP7	0.29	0.10	0.083
ADX94407.1	OBP11	0.23	0	0
ADX94410.1	OBP14	0.12	0.18	0
ACI30690.1	OBP15^*d*^	0.060	0	0.031^*e*^
ADX94402.1	OBP6	0.047^*e*^	0	0
ADX94400.1	OBP4^*d*^	0	0	0.031

^*a*^OBP names from Gotzek et al. (Gotzek et al. 2011).

^*b*^Quantification from exponentially multiplied protein abundance index (emPAI), calculated from mass spectrometry data (Ishihama et al. 2005) and normalized to the emPAI values of glyceraldehyde 3-phosphate dehydrogenase (GAPDH).

^*c*^Also known as Gp-9.

^*d*^Genes located on social chromosome (Pracana et al. 2017).

^*e*^Only one peptide detected.

#### Venom Allergens

Fire ant venom allergen 2 (Sol i 2) was detected in worker and male antennae and in worker tibiae (Supp Table 3 [online only]). This protein was first discovered in *S. invicta* venom (Hoffman et al. 1988, Hoffman 1993), and it is in the poison sac and poison gland (Das et al. 2018). Fire ant workers spray venom in the brood chamber of the nest, probably as an antibiotic, and it is also used by workers as a defensive spray (Obin and Vander Meer 1985). Thus, it is expected that venom allergens would be found on the cuticular surface of colony members. It is reasonable to assume that most of the Sol i 2 detected in the antenna and tibia samples is derived from poison sac venom. However, the normalized amounts of the minor isoform of Sol i 2, venom protein Sol i 4.02, that was detected on the male antenna, and venom protein Sol i 4.01, that was detected on the worker tibia (Supp Table S3 [online only]), are substantially higher than the amounts of these proteins previously detected in extracts of the worker poison sacs (Das et al. 2018).

#### Chemosensory Proteins

Fourteen of the 21 known *S. invicta* chemosensory proteins (CSPs) (Kulmuni et al. 2013) were detected in antennae and tibiae (Table 2). Eleven were detected in worker antennae. Seven of the same proteins were detected in the male antennae, and seven were detected in the tibiae. One additional CSP was detected only in the male antennae, and two were detected only in tibiae. The seven tibial CSPs that were also found in antennae probably are not involved in chemoreception or are multifunctional proteins. The four worker and four male CSPs that are exclusively antennal presumably are involved in odorant reception. Three of the CSPs expressed from genes on the social chromosome (Pracana et al. 2017) were detected: one that was in antennae and tibiae, one that was only in male antennae, and one that was only in tibiae.

**Table 2.** Chemosensory proteins

NCBI accession number	Name^*a*^	Worker antenna^*b*^	Male antenna^*b*^	Worker tibia^*b*^
ACJ64059.1	CSP12^*c*^	1.9	0.69	0.083^*d*^
XP_011160228.1	CSP16	1.9	0	0.23
AAV91325.1	CSP19	1.2	1.8	0
XP_011160273.1	CSP7	0.87	0	0
ACJ64057.1	CSP9	0.45	0.69	0.083
XP_011160926.1	CSP17	0.37	0	0.11
XP_011168346.1	CSP18	0.35	0.85	0.18
XP_011163783.1	CSP1	0.29	0.10	0
AKP92835.1	CSP3	0.25	0.57	0.071
XP_011160226.1	CSP11	0.13	1.1	0
XP_011160224.1	CSP21	0.097	0	0.050
AKP92833.1	CSP13^*c*^	0	0.12^*d*^	0
ACJ64056.1	CSP8^*c*^	0	0	0.18
XP_011160225.1	CSP15	0	0	0.11

^*a*^CSP names from Kulmuni et al. (Kulmuni et al. 2013).

^*b*^Quantification from exponentially multiplied protein abundance index (emPAI), calculated from mass spectrometry data (Ishihama et al. 2005) and normalized to the emPAI values of glyceraldehyde 3-phosphate dehydrogenase (GAPDH).

^*c*^Genes located on social chromosome (Pracana et al. 2017).

^*d*^Only one peptide detected.

#### Other Ligand-Binding/Transport Proteins

In addition to OBPs and CSPs, three other protein families with potential ligand-binding/transport activity related to chemical signal detection were identified. We found ten tubular lipid-binding (TULIP) proteins (Kopec et al. 2011, Wong and Levine 2017) (Table 3), using either genome annotations (‘juvenile hormone-binding protein’ or ‘circadian clock-controlled protein’), or 3D homology modeling of sequences marked ‘uncharacterized protein’. Five different TULIP proteins were found in worker antennae, seven in male antennae, and two in worker tibiae. Of these proteins, three were uniquely expressed in worker antennae and four were uniquely expressed in male antennae. We also found three lipocalin sequences, identified either by annotation (‘apolipoprotein D’) or by 3D homology modeling. The antennae and tibiae each had two different lipocalin sequences, but only the worker antennae expressed a sequence not found in tibiae. Finally, two Niemann–Pick C2 sequences were found, one expressed in all three appendages and one expressed only in worker antennae.

**Table 3.** Other binding/transport proteins

NCBI accession number	Family	Worker antenna^*a*^	Male antenna^*a*^	Worker tibia^*a*^
XP_011171049.1	TULIP^*b*^	0.49	0	0
XP_011171041.1	TULIP	0.35	0	0
XP_011171091.1	TULIP	0.35	0	0
XP_025986354.1	TULIP	0.20	0.28	0.11
XP_011171044.1	TULIP	0.06^*d*^	0.04	0
XP_011171046.1	TULIP	0	0.56	0
XP_011171043.1	TULIP	0	0.10	0.04
XP_011169150.1	TULIP	0	0.05^*d*^	0
XP_011171045.1	TULIP	0	0.05^*d*^	0
XP_011171034.1	TULIP	0	0.04^*d*^	0
XP_011162593.1	lipocalin	0.16	0.05	0.08
XP_011160582.1	lipocalin	0.08^*d*^	0	0
XP_011166366.1	lipocalin	0	0.05	0.04^*d*^
XP_011161896.1	NPC2^*c*^	0.29	0.69	0.035^*d*^
XP_011161897.1	NPC2	0.31	0	0

^*a*^Quantification from exponentially multiplied protein abundance index (emPAI), calculated from mass spectrometry data (Ishihama et al. 2005) and normalized to the emPAI values of glyceraldehyde 3-phosphate dehydrogenase (GAPDH).

^*b*^TULIP, tubular lipid-binding protein.

^*c*^NPC2, Niemann–Pick C2 protein.

^*d*^Only one peptide detected.

### Odorant/Pheromone Degrading Enzymes

#### Cytochrome P450

Twenty-nine different cytochrome P450 sequences were detected in extracts from worker antennae, and 23 P450 sequences were detected in male antennae (Table 4). Twenty-five of these P450 sequences were antennal and were not found in worker tibiae: 10 exclusively in worker antennae, five exclusively in male antennae, and 10 in both. These 25 P450s are likely to include enzymes involved in deactivation of odorant and pheromone molecules. In addition, 10 P450s were found in both antennae and tibiae, and 6 were found exclusively in tibiae. These 16 P450s are likely to include enzymes involved in metablolic reactions unrelated to olfaction. Of the 10 most abundant P450s in worker antennae, six were also found in tibiae; and of the 10 most abundant P450s in male antennae, seven were also found in tibiae. Thus, it appears that generally, the P450s involved in chemosensory deactivation are expressed at lower levels than metabolic P450s.

**Table 4.** Cytochrome P450

NCBI accession number	Class^*a*^	Type^*b*^	Worker antenna^*c*^	Male antenna^*c*^	Worker tibia^c^
XP_025990771.1	4	4C1	2.3	0.40	0.42
XP_011169811.1	4	4C1	1.9	0.33	0.52
XP_011164432.1	3	6a14^*d*^	0.85	0.33	0
XP_025990773.1	4	4C1	0.77	0.33	0.041
XP_025995732.1	3	6a14	0.71	0.25	0.088
XP_025995731.1	3	6a14	0.71	0	0.088
XP_025986987.1	3	6k1	0.32	0.037	0
XP_025986982.1	3	6k1	0.31	0.068	0
XP_011162032.1	3	6k1	0.30	0.065	0
XP_011172133.1	3	6j1	0.26	0.099	0.11
XP_025986058.1	4	4C1	0.23	0	0.026
XP_025991592.1	4	4c21	0.20	0	0
XP_025986433.1	3	9e2	0.15	0.049	0
XP_011175444.1	3	9e2	0.15	0.022	0
XP_011158950.2	3	6k1	0.12	0	0
XP_011155385.1	3	9e2	0.11	0.036	0.038
XP_011172783.1	3	9e2	0.075	0.063	0.085
AAQ90477.1	4	4C1^*d*^	0.075	0.036	0
XP_025986432.1	3	9e2	0.075	0	0
XP_011175440.1	3	9e2	0.069	0.036	0
XP_011158271.1	3	9e2	0.069	0.036	0
XP_025987661.1	3	6a1	0.048	0.032	0
XP_025988558.1	2	304a1	0.037^*e*^	0	0
XP_011172178.1	3	6j1	0.037^*e*^	0	0
XP_011164533.1	3	6k1	0.037^*e*^	0	0
XP_025989949.1	4	4C1	0.032^*e*^	0	0
XP_011167674.2	3	6a13	-^*f*^	-^*e*^	0
XP_011172542.1	4	4C1	-^*f*^	0	0
XP_025994582.1	3	6a20	-^*f*^	0	0
XP_025992592.1	3	6a14	0	0.13	0.11
XP_011172813.1	3	9e2	0	0.049	0
XP_025996422.1	4	4C1	0	0.026	0
XP_011166535.1	3	9e2	0	0.024	0
XP_011160035.1	3	9e2	0	0.012^*e*^	0
XP_025993629.1	2	305a1	0	0.012^*e*^	0
AJW31562.1	4	4C1	0	0	0.085
XP_011164433.2	3	6a14	0	0	0.062
AJW31561.1	4	4G15	0	0	0.059
XP_025986515.1	4	4C1^*d*^	0	0	0.026
XP_025986504.1	3	9e	0	0	0.018
XP_011164781.1	mito	12A2	0	0	0.018^*e*^

^*a*^See Feyereisen (Feyereisen 2012).

^*b*^Assigned using NCBI annotation or by sequence alignment (see note ^*d*^).

^*c*^Quantification from exponentially multiplied protein abundance index (emPAI), calculated from mass spectrometry data by Mascot and normalized to the emPAI values of glyceraldehyde 3-phosphate dehydrogenase (GAPDH).

^*d*^Assigned by sequence alignment rather than annotation.

^*e*^Only one peptide detected.

^*f*^Full sequence not known, so emPAI cannot be calculated.

Four sequences were detected that correspond to genes coding for tandem P450 sequences (i.e., two coding sequences connected by a short length of nucleotides lacking a stop codon and a new start codon). Two of the proteins were found in both worker and male antennae but not in tibiae, and two were found in tibiae but not antennae. The most abundantly expressed tandem P450, XP_025986982.1, was found in antennae. For this protein in workers, 13 out of 15 tryptic peptides detected by mass spectrometry were in the N-terminal half of the sequence, and in males 9 of 12 detected peptides were in the N-terminal half. This strongly suggests that the two halves correspond to separate proteins. Between the two halves, there may be some missing genomic nucleotides that contain termination and start codons, or there could be transcriptional, post-transcriptional or post-translational processing that results in separate proteins. For the other three tandem P450s, the number of detected peptides was too small to draw conclusions about whether the halves were separate.

#### Other Oxidases, Esterases, and Transferases

Other oxidases, esterases, and transferases were detected that may be involved in odorant or pheromone deactivation. Worker antennae had 22 of these enzymes (Table 5), of which 14 were also present in male antennae and 11 in worker tibiae. Male antennae had three additional enzymes in this group that were not in worker antennae, two of which were also present in worker tibiae, and there were nine of the enzymes detected only in worker tibiae. Twelve of the deactivation enzymes were detected only in antennae, including five that were found in both worker and male antennae, one found only in male antennae, and six that were found only in worker antennae. The antenna-specific enzymes included glutathione S-transferases, UDP-glucuronosyltransferases, retinol dehydrongenase, carboxylesterases, and xanthine dehydrogenase. The sequences in Table 5 annotated ‘xanthine dehydrogenase’ are probably aldehyde oxidases, because they lack the conserved tyrosine at the NAD^+^ binding site in xanthine dehydrogenase (Merlin et al. 2005). The sequences annotated ‘retinol dehydrogenase’ are probably short-chain dehydrogenases, and they may perform functions similar to the previously described antennal aldehyde reductases (Guo et al. 2014).

**Table 5.** Other putative odorant/pheromone-degrading enzymes

NCBI accession number	Enzyme^*a*^	Worker antenna^*b*^	Male antenna^*b*^	Worker tibia^*b*^
XP_011165644.1	GST^*c*^	0.74	0.39	0.49
XP_025993326.1	xanthine dehyd.^*d*^	0.63	0.38	0.017
XP_025992003.1	GST	0.57	0.45	0.055
XP_025996377.1	GST 1-like	0.55	0	0.069
XP_011158662.1	GST 1	0.29	0.63	0.21
XP_011165884.1	xanthine dehyd. 1-like	0.36	0.29	0.017
XP_025989394.1	UDP-GT^*e*^ 2B31-like	0.40	0.15	0
XP_011159399.1	retinol dehyd.^*f*^ 12	0.25	0.53	0.10
XP_011159136.1	GST theta-3 X1	0.20	0.18	0.075
ABA39530.1	GST	1.8	0.98	0.94
XP_025987486.1	UDP-GT1-9	0.25	0.063	0.062
XP_025990737.1	UDP-GT 2B1-like X2	0.24	0.11	0
XP_025987060.1	retinol dehyd. 12	0.16	0	0
XP_025995400.1	UDP-GT 2B20	0.20	0.031	0.024^*g*^
XP_011160692.1	esterase FE4 X2	0.16	0.022	0
XP_025995399.1	UDP-GT 2C1-like	0.14^*g*^	0	0
XP_025994825.1	JH^*h*^ esterase-like	0.14	0	0.051^*g*^
XP_011165649.2	GST	0.097	0	0
XP_025991890.1	UDP-GT 2B31	0.11	0	0
XP_011163179.1	esterase E4 X1	0.11	0	0
XP_011168182.1	GST 1 X2	0.052^*g*^	0.39	0
XP_025989254.1	xanthine dehyd. X1	0.0068^*g*^	0.0043	0
XP_011164602.1	esterase FE4-like	0	0.10	0.018^*g*^
XP_011165643.1	GST-like	0	0.15	0
XP_011162023.1	carboxylesterase-6	0	0.27	0.062
XP_025990047.1	retinal dehyd. 1	0	0	0.13
XP_011165650.1	GST-like	0	0	0.075
XP_011162756.1	retinol dehyd. 11	0	0	0.045
XP_011168216.2	UDP-GT 2B31	0	0	0.038
XP_011165649.2	GST	0	0	0.024^*g*^
XP_011164603.1	retinol dehyd. 14	0	0	0.022^*g*^
XP_011170697.1	retinol dehyd. 13	0	0	0.022^*g*^
XP_011171057.1	UDP-GT 2B31	0	0	0.021^*g*^

^*a*^Assigned from annotation.

^*b*^Quantification from exponentially multiplied protein abundance index (emPAI), calculated from mass spectrometry data by Mascot and normalized to the emPAI values of glyceraldehyde 3-phosphate dehydrogenase (GAPDH).

^*c*^GST, glutathione-S-transferase.

^*d*^Xanthine dehydrogenase.

^*e*^UDP-GT, UDP-glucuronosyltransferase.

^*f*^Retinol dehydrogenase.

^*g*^Only one peptide detected.

^*h*^JH, juvenile hormone.

### Olfactory Receptors

No odor-specific olfactory receptors (ORs), ionotropic glutamate-type olfactory receptors (IRs), or gustatory receptors (GRs) were detected in the antennae, presumably because the expression levels of these receptors are below the mass spectrometer detection threshold. In the case of ORs, the receptors are heteromers in which each odor-specific receptor subunit is paired 1:1 with a common co-receptor subunit known as Orco (Larsson et al. 2004, Vosshall and Hansson 2011, Butterwick et al. 2018). Thus, the amount of Orco should equal the total amount of all odor-specific receptors expressed in the antenna, and it can be used as a proxy for the OR content (neglecting Orco homomers). Orco was detected in worker and male antennae, but not in tibiae. The ratio of the abundance of Orco in the worker antenna to the male antenna was approximately 5:1 (Table 6). Two IR subunits (named after *Drosophila melanogaster* IR8a and IR25a) have been described as having coreceptor-like function when combined with other IRs (Abuin et al. 2011), and sequences corresponding to these are known in *S. invicta* (XP_025995684.1 and XP_011156270.1; both are annotated as IR25a-like, but XP_025995684.1 appears to be the IR8a-like sequence). However, the total number of annotated IR genes in *S. invicta* is low: fewer than 40 IRs, compared with more than 200 annotated ORs. At this low level, even the proxy coreceptors are not detectable by mass spectrometry. Similarly, fewer than 30 *S. invicta* GR sequences have been annotated, and GRs are not known to have a coreceptor.

**Table 6.** Orco and Orco complexes

Whole antenna	Orco^*a*^	W/M^*c*^ ratio	SNMP^*a*^	W/M^*e*^ ratio	SNMP/Orco ratio		
Worker, total	3.1		5.0		1.6		
		4.8		0.78			
Male, total	0.64		6.4		10		
Native gel sections	Orco^*b*^	Hi/Lo^*d*^ ratio	SNMP^*b*^	Hi/Lo^*f*^ ratio	SNMP/Orco ratio	P450^*g*^	P450/orco ratio
0.4–0.6 MDa dilute	0.40		0.91		2.3	1.4^*h*^	3.5
		1.3		2.3		0.26^*i*^	0.66
0.2–0.35 MDa, dilute	0.30		0.40		1.3	0.29^*h*^	0.95
						0.19^*i*^	0.63
0.4–0.6 MDa, conc.	0.51		1.6		3.2	1.8^*h*^	3.5
		1.2		1.2		1.1^*i*^	2.1
0.2–0.35 MDa, conc.	0.44		1.3		3.0	0.37^*h*^	0.83
						0.39^*i*^	0.88

^*a*^Quantification from exponentially multiplied protein abundance index (emPAI), calculated from mass spectrometry data (Ishihama et al. 2005), normalized to the emPAI values of glyceraldehyde 3-phosphate dehydrogenase (GAPDH).

^*b*^Quantification from emPAI, normalized to trypsin autolysis peptides.

^*c*^Ratio of Orco in workers to males.

^*d*^Ratio of Orco in 0.4–0.6 mDa gel section to 0.2–0.35 MDa gel section.

^*e*^Ratio of SNMP1 in workers to SNMP1 in males.

^*f*^Ratio of SNMP1 in 0.4–0.6 mDa gel section to 0.2–0.35 MDa gel section.

^*g*^Quantification from emPAI calculated from mass spectrometry data by Mascot, normalized to trypsin autolysis peptides.

^*h*^Data for XP_011164432.1.

^*i*^Data for XP_025986987.1.

Another possible olfactory receptor subunit is sensory neuron membrane protein (SNMP) (Jin et al. 2008, Vogt et al. 2009, German et al. 2013, Gomez-Diaz et al. 2016). SNMP1 was detected in both worker and male antennae, but not in tibiae. The worker:male ratio in antennae was approximately 1:1 (Table 6) Because of the much larger amount of Orco in workers, the SNMP:Orco ratios were about 2:1 in workers and 10:1 in males (Table 6). This difference may be related to the involvement of SNMP in setting the olfactory signal response rate (Discussion).

In order to identify possible olfactory receptor complexes, we also analyzed dodecyl maltoside extracts of worker antennae by blue native gel electrophoresis. The extraction conditions were selected to preserve native oligomeric complexes of integral membrane proteins (Wittig and Schagger 2009, Wittig et al. 2010). We analyzed proteins found in two regions of the electrophoresis gels: approximately 200–350 kD and 400–600 kD. The lower molecular weight region would contain heteromers of Orco and OR subunits (‘tetramers’), containing two subunits of each (Orco_2_OR_2_). The higher molecular weight region would contain higher molecular weight heteromers (‘octamers’, e.g., Orco_4_OR_4_). The results (Table 6) show that Orco is present in both the lower and higher molecular weight regions, consistent with the possibility that both Orco_2_OR_2_ and Orco_4_OR_4_ complexes are formed. However, the higher molecular weight complex might contain Orco_2_OR_2_ complexed with other proteins. For example, in addition to Orco, substantial amounts of SNMP were also detected in both regions of the native gels. Our results cannot distinguish which proteins are associated into protein complexes, so it is not clear whether the detected SNMP is self-associated (e.g., SNMP_4_ or SNMP_8_) (Thorne et al. 1997) or part of a complex containing Orco and OR subunits (e.g., Orco_2_OR_2_SNMP_2_ or Orco_2_OR_2_SNMP_4_) (German et al. 2013). To gain some insight into which subunits may be associated, we compared native electrophoresis samples at two different protein concentrations, differing by a factor of 3. Analytical size separations based on transport, such as gel electrophoresis, usually will disturb subunit equilibria. However, the blue native gel procedure involves addition of Coomassie G250 prior to electrophoresis to displace the detergent used in protein complex extraction and add negative charges to the complexes (Heuberger et al. 2002). Detergent-solubilized membrane proteins are known to exchange subunits, probably by a micelle collision mechanism (Lai and Renthal 2013). However, it is likely that after the native gel Coomassie displacement procedure, negative charge repulsion between colliding complexes would suppress subunit exchange, thus locking-in the oligomeric subunit distribution that had been established by equilibrium in detergent. Therefore, we expect that a comparison of native gels of samples at two different total protein concentrations will show any differences in the subunit distribution between the lower and higher molecular weight regions that reflect concentration-dependent shifts in the subunit equilibria. We found that the high/low molecular weight ratio of Orco (e.g., total amount of Orco in Orco_4_OR_4_ compared to Orco_2_OR_2_) is about 1, both in the dilute samples and in the concentrated samples (Table 6). This indicates that Orco-containing complexes have very high affinities and do not dissociate in the concentration range that we are sampling (estimated as 10–100 nM). In the lower molecular weight gel regions, the relative amounts of SNMP increase in the concentrated protein samples, suggesting concentration-dependent aggregation of SNMP monomers or dimers into tetramers.

We also detected some olfactory-related binding proteins and deactivation enzymes in the native gel segments corresponding to the Orco/OR ‘tetramers’ and ‘octamers’. Twenty P450 sequences were detected in these regions of the native gels. Of these proteins, 8 were sequences we also found in sodium dodecyl sulfate (SDS) extracts of antennae but not tibiae. Only two were present in both the lower and higher molecular weight gel segments of both the concentrated and dilute samples: XP_011164432.1 and XP_025986987.1. The ratios to Orco, shown in Table 6, indicate that both are present at concentrations comparable to or higher than Orco. Whether they form complexes with Orco/OR cannot be determined from these data, in part because P450s can form homomeric complexes in the same molecular weight ranges as the gel segments we analyzed (Reed and Backes 2017). We also detected CSP19 (AAV91325.1) in both the low and high molecular weight regions of the dilute samples and in the low molecular weight region of the concentrated sample. The amounts detected were too low for meaningful calculations of stoichiometry. Although we previously found that CSP19 self-associates to form dimers (Gonzalez et al. 2009), it is very unlikely that this small protein could self-associate to form aggregates having molecular weights above 200 kD. Therefore, its presence in the 200–600 kD region of the native gels suggests the possible formation of strong associations with olfactory receptors or some other protein complex.

## Discussion

Chemical signaling is a significant communication channel in the social organization of ant colonies. Olfactory receptors and contact chemoreceptors are supported by a variety of chemical transporters and deactivation enzymes in chemosensory tissues. Transporters such as odorant-binding proteins (OBPs) and chemosensory proteins (CSPs) and deactivation enzymes such as cytochrome P450s belong to large gene families involved in both chemosensory and non-chemosensory activities. This complicates the study of chemosensory support protein function. We speculated that by comparing the antennal proteomes of fire ant (*S. invicta*) workers and males with the proteome of the tibia, a non-chemosensory appendage, that we would be able to identify probable non-chemosensory support proteins, thereby indicating which support proteins are likely to be essential for odorant transport and deactivation.

### GO Analysis

Antennae were found to have many proteins classified with Biological Process GO terms related to actin polymerization/depolymerization, but these GO terms were not found in tibiae (Supp Table 2 [online only]). More GO terms related to actin polymerization/depolymerization were found in male antennae than in worker antennae. This difference may be related to the differences in sensilla morphology in males and workers. Males have many more sensillae on their antennae than workers, and they are mostly *sensilla tricodea curvata* (Renthal et al. 2003). One interpretation of the enrichment of GO terms relating to actin polymerization/depolymerization in male antennae is that an actin-dependent process is important in the biological function of *sensilla tricodea curvata*.

### Transport Proteins

We found 16 antenna-specific transport proteins in workers and 13 in males (Tables 1–3). Although antennal OBPs have been assumed to be involved in olfaction, recent experiments indicate that many antennal OBPs are not necessary for odorant reception (Larter et al. 2016, Xiao et al. 2019). Also, one antennal OBP was recently suggested to be involved in carrying odorants away from receptors for deactivation (Scheuermann and Smith 2019). One protein we detected in both antennae and tibiae, OBP3 (also known as Gp-9), is present in hemolymph (Krieger and Ross 2002), so it is not possible from our whole-antenna analysis to know whether Gp-9 is also expressed in sensillar lymph. There is evidence that Gp-9 has a role in chemosensory reception. *Gp-9* has two different alleles (*Gp-9B* and *Gp-9b*), and heterozygous workers can detect a transferable substance on the cuticle of homozygous *Gp-9B* queens (Keller and Ross 1998). We found Gp-9B at very high levels in tibiae, and also it was the most abundant OBP in antennae. Gp-9b was detected at very low levels in worker antennae (less than 1% of Gp-9B), and it was not detected in male antennae or tibiae. This is consistent with the very wide range of worker *Gp-9b* allele found in different polygyne colonies (Gotzek and Ross 2008, Leal and Ishida 2008). According to a detailed genotype analysis, the *Gp-9b* allele is frequently carried by male fire ants (Fritz et al. 2006). However, Gp-9 expression in males was reported to be 12-fold lower than workers (Liu and Zhang 2004), which is close to the 11-fold ratio for Gp-9B we found (Table 1). If Gp-9b were present at this level in male antennae from our colonies, it would not be detectable by mass spectrometry.

We detected venom proteins in the antennal and tibial proteomes. The ratios of the Sol i 2 and Sol i 4 isoforms differ in male antennae and tibiae compared with venom sacs (Supp Table S2 [online only]). This raises the possibility that some of the venom proteins are expressed in these appendages as well as the venom sac. The three-dimensional structure of venom allergen 2 (Sol i 2) resembles OBPs (Borer et al. 2012). Sol i 4 has a similar sequence to Sol i 2, so it probably also has an OBP-like structure. It was suggested that these proteins may be involved in pheromone delivery (Borer et al. 2012, Das et al. 2018). Thus, it is possible that the Sol i 2 minor isoform and Sol i 4.02 are used as OBPs in chemosensory reception by male fire ants. Also, Sol i 4.01 could be involved in secretion from worker tibial glands (Billen 2009). Alternatively, the amounts of these minor forms expressed in venom sacs may be variable between colonies: the antennae, tibia, and venom sacs in the Supp Table S2 (online only) data were obtained from different colonies.

CSP19 previously was called SinvCSP (Leal and Ishida 2008) or SiCSP1 (Gonzalez et al. 2009), and it was identified as the most abundant chemosensory protein in the *S. invicta* antenna. Surprisingly, two other highly abundant antennal CSPs (CSP 7 and CSP16) were not detected in the earlier analyses. Although it is possible that these proteins were absent because of the variability between different colonies, the difference is more likely due to the methods used in the previous work. Mascot software could not have identified CSP7 in 2008 because its sequence was not in the NCBI database at that time. CSP7 and CSP16 probably could not have been detected by direct Edman sequencing without pyroglutamase treatment, because both have N-terminal glutamine, which likely would have cyclicized into a blocked N-terminal pyroglutamate. We previously speculated that CSP19 carries nestmate recognition signals, by analogy with the major CSP in *Campanotus japonicus* antennae (Ozaki et al. 2005). However, we found that recombinant CSP19 does not bind to cuticular hydrocarbons (Gonzalez et al. 2009), raising the question of whether *S. invicta* might use signals other than cuticular hydrocarbons for nestmate recognition. Now that we have found that the *S. invicta* antenna contains CSP7, a close sequence homolog of the *Campanotus japonicus* cuticular hydrocarbon-binding CSP, the question of the *S. invicta* nestmate recognition signals should be further examined.

### Deactivation Enzymes

We found 20 antenna-specific cytochrome P450 proteins in workers and 16 in males, suggesting a possible widespread role for P450s in fire ant olfaction (Table 4). Studies of specific odorant and pheromone deactivation enzymes previously showed the involvement of P450s (Wojtasek and Leal 1999, Maibeche-Coisne et al. 2004). However, a transcriptome analysis of 57 *D. melanogaster* P450s found that nearly all were expressed in other tissues (Younus et al. 2014), so it is not clear how many of the *S. invicta* P450s that are in antennae but not in tibiae are olfaction-related. For example, we detected AAQ90477.1 at low levels in worker and male antennae but not in tibiae, yet mRNA coding for this P450 (CYP4AB2) was previously found at very high levels in mRNA extracts of whole workers (Liu and Zhang 2004).

Previous studies of insect P450s showed large phylogenetic expansions of species-specific P450s (Feyereisen 2012). Thus, we examined a phylogenetic tree of hymenoptera P450 sequences from *S. invicta* compared with P450 sequences of the non-social hymeropteran, *Nasonia vitripennis* (Oakeshott et al. 2010), to detect P450 sequences that may have expanded specifically to deactivate chemical signals related to social interactions. In a combined cladogram of 125 *S. invicta* P450s and 87 *N. vitripennis* P450s, we marked the locations of *S. invicta* P450s that were antennal and tibial proteins from Table 4 (Supp Fig. S1 [online only]). The sequences of mitochondrial and CYP clan 2 P450s showed mixing of *S. invicta* and *N. vitripennis* sequences within branches. In CYP clan 3, the 9e2 type proteins showed several separate *S. invicta* and *N. vitripennis* expansions, each containing 7 to 10 sequences. Within the *S. invicta* 9e2 expansions, eight of nine detected sequences were exclusively antennal, suggesting that these P450s might be used by fire ants to deactivate socially relevant chemical signals. In contrast, the 6k1 type proteins of CYP clan 3 show *S. invicta* and *N. vitripennis* branch mixing in the cladogram. Although all 5 of the detected sequences (Table 4) of the 6k1 type are exclusively antennal, the mixing between *S. invicta* and *N. vitripennis* branches suggests the olfactory substrates might be environmental rather than social cues. Although type 6j1 and 6a14 P450s form separate *S. invicta* and *N. vitripennis* expansions, four of five 6a14 and one of two 6j1 type proteins are expressed in the tibia, suggesting these may not be involved in degradation of chemosensory signals. For CYP clan 4, one branch has separate *S. invicta* and *N. vitripennis* 4c1 type expansions, with three of the four detected *S. invicta* proteins expressed in the antenna. A second large branch has a mixture of clan 4 types, and within the branches both *S. invicta* and *N. vitripennis* sequences are intermixed, with five of six detected sequences expressed in tibia, suggesting non-chemosensory functions.

**Fig. 2.**
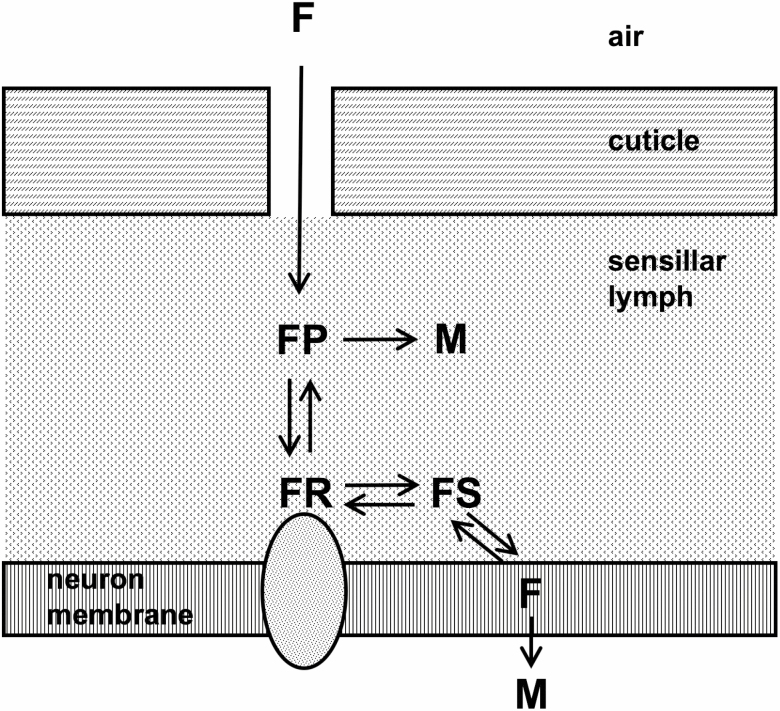
Model for pheromone deactivation. Modified from Kaissling (Kaissling 2009, 2013). Upper rectangles: cuticle, showing pore leading from air to antennal lymph. Lower rectangle: lipid bilayer of olfactory receptor neuron membrane with olfactory receptor shown as embedded oval. F: pheromone or odorant; FP: pheromone or odorant bound to odorant-binding protein or other transport protein; M: metabolite of pheromone or odorant after deactivation; FR: pheromone or odorant bound to extracellular domain of olfactory receptor; FS: complex between pheromone or odorant and SNMP1 or other receptor-to-membrane transporter. Deactivation enzymes catalyzing the FP to M and F to M steps are not shown.

A relatively large amount of SNMP was detected in male antennae, approximately five times more than in workers (Table 6). Previous studies showed that SNMP has a role in deactivation of pheromone responses, because the olfactory receptor neuron’s response was prolonged when an anti-SNMP antibody was present (Jin et al. 2008). A higher rate of deactivation would allow a faster response to changes in pheromone levels, and response speed may be necessary for a male during a mating flight, compared with a worker that crawls on the ground. Placing SNMP in the deactivation step of the olfaction signaling pathway contrasts with the model proposed by Gomez-Diaz et al. (Gomez-Diaz et al. 2016), in which SNMP transfers pheromones from binding proteins to the pheromone receptor. Positioning SNMP activity on the deactivation side simplifies certain aspects of insect olfaction models. SNMP might function to transfer hydrophobic pheromones from their OR binding site to the hydrocarbon region of the membrane lipid bilayer (Fig. 2), via the interior tunnel known to occur in CD36 (Neculai et al. 2013) and modeled in SNMP (Gomez-Diaz et al. 2016). For hydrophobic substances that are water-insoluble, essentially, there would be no free pheromone in the aqueous medium. Placing SNMP on the deactivation pathway of chemosensory reception also simplifies the mechanism for the involvement of cytochrome P450 in deactivation. Membrane-bound cytochrome P450 can access substrates that are in the lipid bilayer (Denisov et al. 2012). If pheromones are delivered to the bilayer after OR reception, it would not be necessary for P450 to be anchored facing the antennal lymph side of the olfactory receptor neuron membrane. Also, there would be no need for the postulated (Maibeche-Coisne et al. 2004) membrane transporter for electrons from cytoplasmic-facing cytochrome P450 reductase in order for P450 to deactivate pheromones.

Analogous to SNMP, receptor-to-membrane transfer of odorants or pheromones may also be a property of some tubular lipid-binding (TULIP) proteins, 8 of which were detected in the antennae of workers and/or males, but not in worker tibia (Table 3). TULIP proteins have been previously detected in chemosensory tissue (Dauwalder et al. 2002, Bohbot and Vogt 2005, Saito et al. 2006, Renthal et al. 2017), but their role in chemosensory reception is unknown. Three-dimensional structures of TULIP proteins show that they contain interior tunnels (Mueller et al. 2010, Fujimoto et al. 2013, Hamiaux et al. 2013), similar to SNMP. In one study, RNAi knockdown of a TULIP protein resulted in a specific change in olfactory sensitivity (Guo et al. 2011).

### Oligomeric Structure of ORs

We detected Orco on native gels at molecular weights between 200 and 350 kD (Table 6). This is consistent with a tetrameric structure (Orco_2_OR_2_) for the olfactory receptor, as proposed by Butterwick et al. (Butterwick et al. 2018). Although the cryoelectron microscopy structure, cross-linking, and mutagenesis results of Butterwick et al. all point to Orco_2_OR_2_ as the active form of the receptor, the native gel data they reported appears to show an octamer, Orco_8_, in reference to water-soluble calibration standards, but a tetramer in reference to one membrane protein standard. Our native gels were calibrated with four bovine mitochondrial membrane protein complexes, which were shown (Wittig et al. 2010) to give accurate molecular weights for integral membrane protein complexes. We also detected Orco between 400 and 600 kD, which is consistent with either an octamer (Orco_4_OR_4_) or a complex between Orco/OR tetramers and additional subunits, such as SNMP.

A *D. melanogaster* Orco interactome was reported by Yu et al. (Yu et al. 2018), based on a pull-down method. No SNMP was detected, but several cytochrome P450s were identified. We found numerous P450s migrating in native gels in the same molecular weight range as Orco. However, it is not clear whether these P450s form complexes with olfactory receptors or are self-associating. The *D. melanogaster* Orco interactome also contained one OBP (Yu et al. 2018). We did not detect any OBPs in the same native gel regions as Orco, but we did find CSP19 (Table 6), indicating the possibility of a complex between it and ORs.

### Receptor and Support Protein Diversity

The ratio of Orco in worker antennae to male antennae is a measure of the relative numbers of olfactory receptors of the two castes. Using a label-free proteomics method, we estimate the ratio to be 5:1 (Table 6). This is in accord with what is known about the relative complexity of colony interactions of ant workers and males (Boomsma et al. 2005, Shik et al. 2013). The diversity of ORs does not necessarily parallel the amount of Orco, which may be skewed in one caste compared with another if the same odor-specific OR gene products are expressed at much higher levels in one caste, or if one caste has a higher level of self-associated Orco. The number of antennal lobe glomeruli is generally considered to be a good estimate for the number of different OR genes expressed. Typical social insect workers are known to have many more glomeruli than males (Arnold et al. 1985, Zube and Rossler 2008, Nakanishi et al. 2010, McKenzie et al. 2016), in contrast to non-social hymenoptera, where the numbers of antennal lobe glomeruli in males and females are about equal (Das and Fadamiro 2013). Considering the high worker-to-male Orco ratio that we measured, and the high worker-to-male antennal lobe glomeruli ratios previously measured in ants, it is surprising that the diversity ratio is so much lower for antenna-specific olfactory binding/transport proteins (Tables 1–3) and deactivation enzymes (Tables 4–5). The ratio of the number of different antennal-specific binding/transport/deactivation proteins in Tables 1–5 for workers compared with males is about 1:1. It is possible that this ratio is distorted by a classification artifact: many of the proteins we have classified as chemosensory support proteins might actually have antenna-specific functions that are unrelated to chemosensory reception. However, it is not clear why there would be more of these in males than workers. A second possible artifact could result from the limited lower range sensitivity of proteome detection by mass spectrometry. For example, workers might express low levels of many more proteins than we detected, or males might express many more non-OR odorant receptors (e.g., in the IR family) than we detected. Neither of these possibilities seems promising, considering the known limited number of additional IRs, OBPs, CSPs, and P450s that have been identified so far in the *S. invicta* genome. There is evidence supporting the similarity of worker and male OBP expression down to low levels. A study of fire ant antennal OBP mRNA (Zhang et al. 2016) shows a similar pattern to our proteomics data in Table 1 for high-level transcripts, but no gap between workers and males appears in the low-level OBP transcripts, which are at about the same levels.

**Fig. 3.**
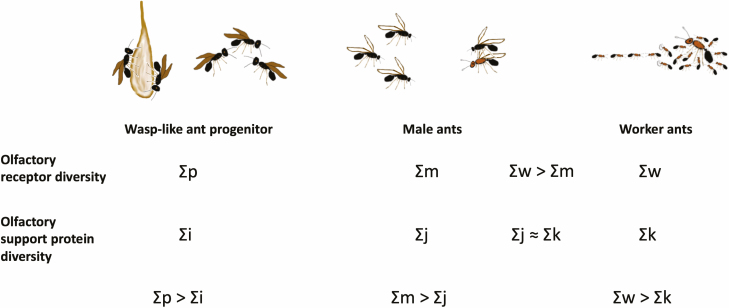
Model for comparing of OR and olfactory support protein diversity. Each olfactory support protein can transport or deactivate groups of substances that belong to a homologous series of organic compounds. In contrast, ORs have much higher specificity, each binding to a very narrow range of substances. The total numbers of different ORs expressed in wasp-like ant progenitors, male ants and male workers are indicated, respectively, as Σp, Σm, and Σw. The total numbers of expressed olfactory support proteins are indicated, respectively, as Σi, Σj, and Σk. Thus, for the full set of chemicals detected by a species or caste, Σp > Σi, Σm > Σj, and Σw > Σk. Due to the reduced chemical signal reception needs of male ants, Σw > Σm. Surprisingly, we found that Σj ≈ Σk. This may reflect the evolutionary origin of the male caste from wasp-like progenitors that performed similar work to females in nest-building, guarding, and queen assessment. Chemical signals with diverse organic functional groups may have been retained for signaling of male ant mating flights and male assessment of female alates, balancing higher protein expression costs against perhaps even higher costs required for evolution of an entirely new signaling scheme having a reduced repertoire of olfactory support proteins, Σj < Σk.

The molecular basis of OR specificity is not yet known, but physiological and behavioral studies show that ORs have a narrower selectivity than binding/transport proteins and deactivation enzymes (Wojtasek et al. 1998, Lautenschlager et al. 2007, Carey et al. 2010, Xu et al. 2012, Katti et al. 2013, Younus et al. 2014). Thus, hundreds of different ORs, each having high specificity for a particular chemical substance, can be supported by many-fold fewer binding/transport proteins and deactivation enzymes, which typically have a wider range of affinity for groups of chemically similar compounds, such as a homologous series of a particular organic functional group. Expression of more copies of a protein per mRNA has a lower fitness cost (Frumkin et al. 2017), so this might seem to favor the evolution of genes for broad-specificity binding/transport proteins and deactivation enzymes. Presumably, as the worker caste evolved, much of the OR complexity was added to support the needs of social interactions. By the same reasoning, the evolution of the male ant caste might be expected to have decreased OR complexity, compared with the wasp-like progenitors of ants. The chemical detection capabilities needed by males in the wasp-like progenitors of ants are unknown, but they may have had behavioral traits similar to solitary or primitively social wasps. In some solitary wasps, male and female tasks overlap in work such as nest building and guarding (Brockmann 1992). For some primitively social wasps, both males and workers have similar needs to assess the quality of female egg-laying fitness: workers in selecting a queen to follow (West-Eberhard 1978), and males for their mating choice. Thus, similar ranges of olfactory receptor diversity may have been required in both males and females in ant progenitors. The chemicals constituting these signals probably covered a relatively wide range of organic functional groups, and this underlying diversity of the chemical repertoire thus would be retained in the expanded signals used by ant colonies as they evolved to higher levels of complexity, where the male caste does no colony work (Fig. 3). Apparently, it was less costly for male ants to retain and express ORs and olfactory support proteins encompassing a diverse set of chemical specificities than for ant colonies to evolve an entirely new chemical coding system having fewer organic functional groups to represent the limited information required by non-working male ants. We expect that the disparity between the diversity of male ant ORs and chemosensory support proteins is more extreme in *S. invicta*, which uses an aerial swarm mating system, than in ants that use a female-calling mating system (Boomsma et al. 2005), which may require more diverse male ORs.

## Data Availability

Thermo raw mass spectra files and Mascot generic format peak lists are publically available in the MassIVE Repository: ftp://massive.ucsd.edu/MSV000085997/.

## Supplementary Material

ieaa118_suppl_Supplementary_Material

## References

[CIT0001] Abuin L, Bargeton B, Ulbrich M H, Isacoff E Y, Kellenberger S, and Benton R. 2011 Functional architecture of olfactory ionotropic glutamate receptors. Neuron. 69: 44–60. 21220098 10.1016/j.neuron.2010.11.042 PMC3050028

[CIT0002] Arnold G, Masson C, and Budharugsa S. 1985 Comparative-study of the antennal lobes and their afferent pathway in the worker bee and the drone *(Apis mellifera)*. Cell Tissue Res. 242: 593–605.

[CIT0003] Billen J 2009 Occurrence and structural organization of the exocrine glands in the legs of ants. Arthropod Struct. Dev. 38: 2–15. 18775512 10.1016/j.asd.2008.08.002

[CIT0004] Bohbot J, and Vogt R G. 2005 Antennal expressed genes of the yellow fever mosquito (*Aedes aegypti* L.); characterization of odorant-binding protein 10 and takeout. Insect Biochem. Mol. Biol. 35: 961–979. 15978998 10.1016/j.ibmb.2005.03.010

[CIT0005] Boomsma J J, Baer B, and Heinze J. 2005 The evolution of male traits in social insects. Annu. Rev. Entomol. 50: 395–420. 15822204 10.1146/annurev.ento.50.071803.130416

[CIT0006] Borer A S, Wassmann P, Schmidt M, Hoffman D R, Zhou J J, Wright C, Schirmer T, and Marković-Housley Z. 2012 Crystal structure of Sol I 2: a major allergen from fire ant venom. J. Mol. Biol. 415: 635–648. 22100449 10.1016/j.jmb.2011.10.009

[CIT0007] Brockmann H J 1992 Male-behavior, courtship and nesting in *Trypoxylon (Trypargilum) monteverdeae* (Hymenoptera, Sphecidae). J. Kansas Entomol. Soc. 65: 66–84.

[CIT0008] Butterwick J A, Del Mármol J, Kim K H, Kahlson M A, Rogow J A, Walz T, and Ruta V. 2018 Cryo-EM structure of the insect olfactory receptor Orco. Nature. 560: 447–452. 30111839 10.1038/s41586-018-0420-8 PMC6129982

[CIT0009] Carey A F, Wang G, Su C Y, Zwiebel L J, and Carlson J R. 2010 Odorant reception in the malaria mosquito *Anopheles gambiae*. Nature. 464: 66–71. 20130575 10.1038/nature08834 PMC2833235

[CIT0010] Cohanim A B, Amsalem E, Saad R, Shoemaker D, and Privman E. 2018 Evolution of olfactory functions on the fire ant social chromosome. Genome Biol. Evol. 10: 2947–2960. 30239696 10.1093/gbe/evy204 PMC6279166

[CIT0011] Das P, and Fadamiro H Y. 2013 Species and sexual differences in antennal lobe architecture and glomerular organization in two parasitoids with different degree of host specificity, *Microplitis croceipes* and *Cotesia marginiventris*. Cell Tissue Res. 352: 227–235. 23420449 10.1007/s00441-013-1568-z

[CIT0012] Das T, Alabi I, Colley M, Yan F, Griffith W, Bach S, Weintraub S T, and Renthal R. 2018 Major venom proteins of the fire ant *Solenopsis invicta*: insights into possible pheromone-binding function from mass spectrometric analysis. Insect Mol. Biol. 27: 505–511. 29656567 10.1111/imb.12388 PMC6188847

[CIT0013] Dauwalder B, Tsujimoto S, Moss J, and Mattox W. 2002 The Drosophila takeout gene is regulated by the somatic sex-determination pathway and affects male courtship behavior. Genes Dev 16: 2879–2892. 12435630 10.1101/gad.1010302 PMC187483

[CIT0014] Denisov I G, Shih A Y, and Sligar S G. 2012 Structural differences between soluble and membrane bound cytochrome P450s. J. Inorg. Biochem. 108: 150–158. 22244217 10.1016/j.jinorgbio.2011.11.026 PMC4190058

[CIT0015] Feyereisen R 2012 Insect CYP Genes and P450 Enzymes, pp. 236–316. *In* Insect molecular biology and biochemistry. Academic Press, London, United Kingdom.

[CIT0016] Fritz G N, Vander Meer R K, and Preston C A. 2006 Selective male mortality in the red imported fire ant, *Solenopsis invicta*. Genetics. 173: 207–213. 16489215 10.1534/genetics.106.056358 PMC1461442

[CIT0017] Frumkin I, Schirman D, Rotman A, Li F, Zahavi L, Mordret E, Asraf O, Wu S, Levy S F, and Pilpel Y. 2017 Gene architectures that minimize cost of gene expression. Mol. Cell 65: 142–153. 27989436 10.1016/j.molcel.2016.11.007 PMC5506554

[CIT0018] Fujimoto Z, Suzuki R, Shiotsuki T, Tsuchiya W, Tase A, Momma M, and Yamazaki T. 2013 Crystal structure of silkworm Bombyx mori JHBP in complex with 2-methyl-2,4-pentanediol: plasticity of JH-binding pocket and ligand-induced conformational change of the second cavity in JHBP. PLoS One 8: e56261. 23437107 10.1371/journal.pone.0056261 PMC3577830

[CIT0019] German P F, van der Poel S, Carraher C, Kralicek A V, and Newcomb R D. 2013 Insights into subunit interactions within the insect olfactory receptor complex using FRET. Insect Biochem. Mol. Biol. 43: 138–145. 23196131 10.1016/j.ibmb.2012.11.002

[CIT0020] Gomez-Diaz C, Bargeton B, Abuin L, Bukar N, Reina J H, Bartoi T, Graf M, Ong H, Ulbrich M H, Masson J F, and Benton R. 2016 A CD36 ectodomain mediates insect pheromone detection via a putative tunnelling mechanism. Nat. Commun. 7: 11866. 27302750 10.1038/ncomms11866 PMC4912623

[CIT0021] González D, Zhao Q, McMahan C, Velasquez D, Haskins W E, Sponsel V, Cassill A, and Renthal R. 2009 The major antennal chemosensory protein of red imported fire ant workers. Insect Mol. Biol. 18: 395–404. 19523071 10.1111/j.1365-2583.2009.00883.x PMC2771726

[CIT0022] Gotzek D, and Ross K G. 2008 Experimental conversion of colony social organization in fire ants *(Solenopsis invicta)*: Worker genotype manipulation in the absence of queen effects. J. Insect Behav. 21: 337–350.

[CIT0023] Gotzek D, Robertson H M, Wurm Y, and Shoemaker D. 2011 Odorant binding proteins of the red imported fire ant, *Solenopsis invicta*: an example of the problems facing the analysis of widely divergent proteins. PLoS One 6: e16289. 21305009 10.1371/journal.pone.0016289 PMC3031547

[CIT0024] Guo W, Wang X, Ma Z, Xue L, Han J, Yu D, and Kang L. 2011 CSP and takeout genes modulate the switch between attraction and repulsion during behavioral phase change in the migratory locust. PLoS Genet. 7: e1001291. 21304893 10.1371/journal.pgen.1001291 PMC3033386

[CIT0025] Guo H, Del Corso A, Huang L Q, Mura U, Pelosi P, and Wang C Z. 2014 Aldehyde reductase activity in the antennae of *Helicoverpa armigera*. Insect Mol. Biol. 23: 330–340. 24580848 10.1111/imb.12084

[CIT0026] Hamiaux C, Basten L, Greenwood D R, Baker E N, and Newcomb R D. 2013 Ligand promiscuity within the internal cavity of *Epiphyas postvittana* Takeout 1 protein. J. Struct. Biol. 182: 259–263. 23563188 10.1016/j.jsb.2013.03.013

[CIT0027] Heuberger E H, Veenhoff L M, Duurkens R H, Friesen R H, and Poolman B. 2002 Oligomeric state of membrane transport proteins analyzed with blue native electrophoresis and analytical ultracentrifugation. J. Mol. Biol. 317: 591–600. 11955011 10.1006/jmbi.2002.5416

[CIT0028] Hoffman D R 1993 Allergens in Hymenoptera venom XXIV: the amino acid sequences of imported fire ant venom allergens Sol i II, Sol i III, and Sol i IV. J. Allergy Clin. Immunol. 91: 71–78. 8423273 10.1016/0091-6749(93)90298-t

[CIT0029] Hoffman D R, Dove D E, and Jacobson R S. 1988 Allergens in Hymenoptera venom. XX. Isolation of four allergens from imported fire ant (*Solenopsis invicta*) venom. J. Allergy Clin. Immunol. 82: 818–827. 3192865 10.1016/0091-6749(88)90084-x

[CIT0030] Ishihama Y, Oda Y, Tabata T, Sato T, Nagasu T, Rappsilber J, and Mann M. 2005 Exponentially modified protein abundance index (emPAI) for estimation of absolute protein amount in proteomics by the number of sequenced peptides per protein. Mol Cell Proteomics 4: 1265–1272. 15958392 10.1074/mcp.M500061-MCP200

[CIT0031] Jin X, Ha T S, and Smith D P. 2008 SNMP is a signaling component required for pheromone sensitivity in Drosophila. Proc. Natl. Acad. Sci. U. S. A. 105: 10996–11001. 18653762 10.1073/pnas.0803309105 PMC2504837

[CIT0032] Jouvenaz D P, Allen G E, Banks W A, and Wojcik D P. 1977 A survey for pathogens of fire ants, *Solenopsis* spp., in the southeastern United States. Fla. Entomol. 60: 275–279.

[CIT0033] Kaissling K E 2009 Olfactory perireceptor and receptor events in moths: a kinetic model revised. J. Comp. Physiol. A. Neuroethol. Sens. Neural. Behav. Physiol. 195: 895–922. 19697043 10.1007/s00359-009-0461-4 PMC2749182

[CIT0034] Kaissling K E 2013 Kinetics of olfactory responses might largely depend on the odorant-receptor interaction and the odorant deactivation postulated for flux detectors. J. Comp. Physiol. A. Neuroethol. Sens. Neural. Behav. Physiol. 199: 879–896. 23563709 10.1007/s00359-013-0812-z

[CIT0035] Katti S, Lokhande N, González D, Cassill A, and Renthal R. 2013 Quantitative analysis of pheromone-binding protein specificity. Insect Mol. Biol. 22: 31–40. 23121132 10.1111/j.1365-2583.2012.01167.x PMC3552018

[CIT0036] Keller L, and Ross K G. 1998 Selfish genes: a green beard in the red fire ant. Nature 394: 573–575.

[CIT0037] Kopec K O, Alva V, and Lupas A N. 2011 Bioinformatics of the TULIP domain superfamily. Biochem. Soc. Trans. 39: 1033–1038. 21787343 10.1042/BST0391033

[CIT0038] Krieger M J, and Ross K G. 2002 Identification of a major gene regulating complex social behavior. Science. 295: 328–332. 11711637 10.1126/science.1065247

[CIT0039] Kulmuni J, Wurm Y, and Pamilo P. 2013 Comparative genomics of chemosensory protein genes reveals rapid evolution and positive selection in ant-specific duplicates. Heredity (Edinb). 110: 538–547. 23403962 10.1038/hdy.2012.122 PMC3656642

[CIT0040] Lai G, and Renthal R. 2013 Integral membrane protein fragment recombination after transfer from nanolipoprotein particles to bicelles. Biochemistry. 52: 9405–9412. 24328096 10.1021/bi401391c

[CIT0041] Larsson M C, Domingos A I, Jones W D, Chiappe M E, Amrein H, and Vosshall L B. 2004 Or83b encodes a broadly expressed odorant receptor essential for Drosophila olfaction. Neuron. 43: 703–714. 15339651 10.1016/j.neuron.2004.08.019

[CIT0042] Larter N K, Sun J S, and Carlson J R. 2016 Organization and function of Drosophila odorant binding proteins. Elife 5: e20242. 10.7554/eLife.20242 PMC5127637 27845621

[CIT0043] Lautenschlager C, Leal W S, and Clardy J. 2007 *Bombyx mori* pheromone-binding protein binding nonpheromone ligands: implications for pheromone recognition. Structure. 15: 1148–1154. 17850754 10.1016/j.str.2007.07.013 PMC2072049

[CIT0044] Leal W S, and Ishida Y. 2008 GP-9s are ubiquitous proteins unlikely involved in olfactory mediation of social organization in the red imported fire ant, *Solenopsis invicta*. PLoS One 3: e3762. 19018280 10.1371/journal.pone.0003762 PMC2582448

[CIT0045] Liu N, and Zhang L. 2004 CYP4AB1, CYP4AB2, and Gp-9 gene overexpression associated with workers of the red imported fire ant, *Solenopsis invicta* Buren. Gene. 327: 81–87. 14960363 10.1016/j.gene.2003.11.002

[CIT0046] Maïbèche-Coisne M, Nikonov A A, Ishida Y, Jacquin-Joly E, and Leal W S. 2004 Pheromone anosmia in a scarab beetle induced by in vivo inhibition of a pheromone-degrading enzyme. Proc. Natl. Acad. Sci. U. S. A. 101: 11459–11464. 15277687 10.1073/pnas.0403537101 PMC509178

[CIT0047] McKenzie S K, Fetter-Pruneda I, Ruta V, and Kronauer D J. 2016 Transcriptomics and neuroanatomy of the clonal raider ant implicate an expanded clade of odorant receptors in chemical communication. Proc. Natl. Acad. Sci. U. S. A. 113: 14091–14096. 27911792 10.1073/pnas.1610800113 PMC5150400

[CIT0048] Merlin C, François M C, Bozzolan F, Pelletier J, Jacquin-Joly E, and Maïbèche-Coisne M. 2005 A new aldehyde oxidase selectively expressed in chemosensory organs of insects. Biochem. Biophys. Res. Commun. 332: 4–10. 15896291 10.1016/j.bbrc.2005.04.084

[CIT0049] Mitchell B K, Itagaki H, and Rivet M P. 1999 Peripheral and central structures involved in insect gustation. Microsc. Res. Tech. 47: 401–415. 10607380 10.1002/(SICI)1097-0029(19991215)47:6<401::AID-JEMT4>3.0.CO;2-7

[CIT0050] Mueller G A, Edwards L L, Aloor J J, Fessler M B, Glesner J, Pomés A, Chapman M D, London R E, and Pedersen L C. 2010 The structure of the dust mite allergen Der p 7 reveals similarities to innate immune proteins. J. Allergy Clin. Immunol. 125: 909–917.e4. 20226507 10.1016/j.jaci.2009.12.016 PMC2885876

[CIT0051] Nakanishi A, Nishino H, Watanabe H, Yokohari F, and Nishikawa M. 2010 Sex-specific antennal sensory system in the ant Camponotus japonicus: glomerular organizations of antennal lobes. J. Comp. Neurol. 518: 2186–2201. 20437523 10.1002/cne.22326

[CIT0052] Neculai D, Schwake M, Ravichandran M, Zunke F, Collins R F, Peters J, Neculai M, Plumb J, Loppnau P, Pizarro J C, et al 2013 Structure of LIMP-2 provides functional insights with implications for SR-BI and CD36. Nature. 504: 172–176. 24162852 10.1038/nature12684

[CIT0053] Newland P L, Rogers S M, Gaaboub I, and Matheson T. 2000 Parallel somatotopic maps of gustatory and mechanosensory neurons in the central nervous system of an insect. J. Comp. Neurol. 425: 82–96. 10940944

[CIT0054] Oakeshott J G, Johnson R M, Berenbaum M R, Ranson H, Cristino A S, and Claudianos C. 2010 Metabolic enzymes associated with xenobiotic and chemosensory responses in Nasonia vitripennis. Insect Mol. Biol. 19(Suppl 1): 147–163. 10.1111/j.1365-2583.2009.00961.x 20167025

[CIT0055] Obin M S, and Vander Meer R K. 1985 Gaster flagging by fire ants (Solenopsis spp.): functional significance of venom dispersal behavior. J. Chem. Ecol. 11: 1757–1768. 24311339 10.1007/BF01012125

[CIT0056] Ozaki M, Wada-Katsumata A, Fujikawa K, Iwasaki M, Yokohari F, Satoji Y, Nisimura T, and Yamaoka R. 2005 Ant nestmate and non-nestmate discrimination by a chemosensory sensillum. Science. 309: 311–314. 15947139 10.1126/science.1105244

[CIT0057] Pelosi P, Iovinella I, Felicioli A, and Dani F R. 2014 Soluble proteins of chemical communication: an overview across arthropods. Front. Physiol. 5: 320. 25221516 10.3389/fphys.2014.00320 PMC4145409

[CIT0058] Perkins D N, Pappin D J, Creasy D M, and Cottrell J S. 1999 Probability-based protein identification by searching sequence databases using mass spectrometry data. Electrophoresis. 20: 3551–3567. 10612281 10.1002/(SICI)1522-2683(19991201)20:18<3551::AID-ELPS3551>3.0.CO;2-2

[CIT0059] Pracana R, Levantis I, Martínez-Ruiz C, Stolle E, Priyam A, and Wurm Y. 2017 Fire ant social chromosomes: differences in number, sequence and expression of odorant binding proteins. Evol. Lett. 1: 199–210. 30283649 10.1002/evl3.22 PMC6121795

[CIT0060] Raudvere U, Kolberg L, Kuzmin I, Arak T, Adler P, Peterson H, and Vilo J. 2019 g:Profiler: a web server for functional enrichment analysis and conversions of gene lists (2019 update). Nucleic Acids Res. 47: W191–W198. 31066453 10.1093/nar/gkz369 PMC6602461

[CIT0061] Reed J R, and Backes W L. 2017 Physical studies of P450-P450 interactions: predicting quaternary structures of P450 complexes in membranes from their x-ray crystal structures. Front. Pharmacol. 8: 28. 28194112 10.3389/fphar.2017.00028 PMC5276844

[CIT0062] Renthal R, Velasquez D, Olmos D, Hampton J, and Wergin W P. 2003 Structure and distribution of antennal sensilla of the red imported fire ant. Micron. 34: 405–413. 14680927 10.1016/S0968-4328(03)00050-7

[CIT0063] Renthal R, Manghnani L, Bernal S, Qu Y, Griffith W P, Lohmeyer K, Guerrero F D, Borges L M F, and Pérez de León A. 2017 The chemosensory appendage proteome of *Amblyomma americanum* (Acari: Ixodidae) reveals putative odorant-binding and other chemoreception-related proteins. Insect Sci. 24: 730–742. 27307202 10.1111/1744-7917.12368

[CIT0064] Saito K, Su Z H, Emi A, Mita K, Takeda M, and Fujiwara Y. 2006 Cloning and expression analysis of takeout/JHBP family genes of silkworm, Bombyx mori. Insect Mol. Biol. 15: 245–251. 16756543 10.1111/j.1365-2583.2006.00612.x

[CIT0065] Scheuermann E A, and Smith D P. 2019 Odor-specific deactivation defects in a drosophila odorant-binding protein mutant. Genetics. 213: 897–909. 31492805 10.1534/genetics.119.302629 PMC6827369

[CIT0066] Shik JZ, Donoso DA and Kaspari M 2013 The life history continuum hypothesis links traits of male ants with life outside the nest. Entomol. Exp. Appl. 149: 99–109.

[CIT0067] Silva J C, Gorenstein M V, Li G Z, Vissers J P, and Geromanos S J. 2006 Absolute quantification of proteins by LCMSE: a virtue of parallel MS acquisition. Mol Cell Proteomics 5: 144–156. 16219938 10.1074/mcp.M500230-MCP200

[CIT0068] Stamatakis A 2014 RAxML version 8: a tool for phylogenetic analysis and post-analysis of large phylogenies. Bioinformatics. 30: 1312–1313. 24451623 10.1093/bioinformatics/btu033 PMC3998144

[CIT0069] Thorne R F, Meldrum C J, Harris S J, Dorahy D J, Shafren D R, Berndt M C, Burns G F, and Gibson P G. 1997 CD36 forms covalently associated dimers and multimers in platelets and transfected COS-7 cells. Biochem. Biophys. Res. Commun. 240: 812–818. 9398651 10.1006/bbrc.1997.7755

[CIT0070] Vogt R G, Miller N E, Litvack R, Fandino R A, Sparks J, Staples J, Friedman R, and Dickens J C. 2009 The insect SNMP gene family. Insect Biochem. Mol. Biol. 39: 448–456. 19364529 10.1016/j.ibmb.2009.03.007

[CIT0071] Vosshall L B, and Hansson B S. 2011 A unified nomenclature system for the insect olfactory coreceptor. Chem. Senses 36: 497–498. 21441366 10.1093/chemse/bjr022

[CIT0072] West-Eberhard M J 1978 Temporary queens in metapolybia wasps: nonreproductive helpers without altruism? Science. 200: 441–443. 17757302 10.1126/science.200.4340.441

[CIT0073] Wittig I, and Schägger H. 2009 Native electrophoretic techniques to identify protein-protein interactions. Proteomics. 9: 5214–5223. 19834896 10.1002/pmic.200900151

[CIT0074] Wittig I, Beckhaus T, Wumaier Z, Karas M, and Schägger H. 2010 Mass estimation of native proteins by blue native electrophoresis: principles and practical hints. Mol Cell Proteomics 9: 2149–2161. 20173216 10.1074/mcp.M900526-MCP200 PMC2953912

[CIT0075] Wojtasek H, and Leal W S. 1999 Degradation of an alkaloid pheromone from the pale-brown chafer, *Phyllopertha diversa* (Coleoptera: Scarabaeidae), by an insect olfactory cytochrome P450. FEBS Lett. 458: 333–336. 10570935 10.1016/s0014-5793(99)01178-3

[CIT0076] Wojtasek H, Hansson B S, and Leal W S. 1998 Attracted or repelled?–a matter of two neurons, one pheromone binding protein, and a chiral center. Biochem. Biophys. Res. Commun. 250: 217–222. 9753610 10.1006/bbrc.1998.9278

[CIT0077] Wong L H, and Levine T P. 2017 Tubular lipid binding proteins (TULIPs) growing everywhere. Biochim. Biophys. Acta. Mol. Cell Res. 1864: 1439–1449. 28554774 10.1016/j.bbamcr.2017.05.019 PMC5507252

[CIT0078] Xiao S, Sun J S, and Carlson J R. 2019 Robust olfactory responses in the absence of odorant binding proteins. Elife 8: e51040. 10.7554/eLife.51040 PMC6814364 31651397

[CIT0079] Xu P, Hooper A M, Pickett J A, and Leal W S. 2012 Specificity determinants of the silkworm moth sex pheromone. PLoS One 7: e44190. 22957053 10.1371/journal.pone.0044190 PMC3434217

[CIT0080] Younus F, Chertemps T, Pearce S L, Pandey G, Bozzolan F, Coppin C W, Russell R J, Maïbèche-Coisne M, and Oakeshott J G. 2014 Identification of candidate odorant degrading gene/enzyme systems in the antennal transcriptome of Drosophila melanogaster. Insect Biochem. Mol. Biol. 53: 30–43. 25038463 10.1016/j.ibmb.2014.07.003

[CIT0081] Yu K E, Kim D H, Kim Y I, Jones W D, and Lee J E. 2018 Mass Spectrometry-Based Screening Platform Reveals Orco Interactome in Drosophila melanogaster. Mol. Cells 41: 150–159. 29429152 10.14348/molcells.2018.2305 PMC5824025

[CIT0082] Zhang W, Wanchoo A, Ortiz-Urquiza A, Xia Y, and Keyhani N O. 2016 Tissue, developmental, and caste-specific expression of odorant binding proteins in a eusocial insect, the red imported fire ant, *Solenopsis invicta*. Sci. Rep. 6: 35452. 27765943 10.1038/srep35452 PMC5073229

[CIT0083] Zube C, and Rössler W. 2008 Caste- and sex-specific adaptations within the olfactory pathway in the brain of the ant Camponotus floridanus. Arthropod Struct. Dev. 37: 469–479. 18621145 10.1016/j.asd.2008.05.004

