# Supplementary material for: Antennal Proteome of the Solenopsis invicta (Hymenoptera: Formicidae): Caste Differences in Olfactory Receptors and Chemosensory Support Proteins
Source: J Insect Sci. 2020 Oct 24;20(5):29. doi: 10.1093/jisesa/ieaa118 (PMC7585320; doi:10.1093/jisesa/ieaa118)
Supplement: ieaa118_suppl_Supplementary_Material [file ieaa118_suppl_supplementary_material.pdf]

Supplementary Material

Antennal Proteome of the Fire Ant Solenopsis invicta: Caste Differences in Olfactory Receptors and Chemosensory Support Proteins

Jaee Shailesh Shah and Robert Renthal

Department of Biology

University of Texas at San Antonio

San Antonio, TX 78249

Table S1. Worker-specific antennal proteins[1]

Accession	Score[2]	emPAI[3]	Description
XP_011162016.1	142	3.03	chromobox protein homolog 1 isoform X2
XP_025988515.1	2010	2.97	Na^+/K^+- ATPase subunit α isoform X4
XP_025988512.1	2069	2.96	Na^+/K^+- ATPase subunit α isoform X1
XP_011161897.1	198	2.68	Niemann-Pick C2 homolog a
XP_011173840.2	67	2.66	polyadenylate-binding protein 2-like
XP_011171049.1	243	1.95	takeout-like
XP_011161680.1	279	1.71	keratin, type I cytoskeletal 14
XP_011168537.1	408	1.29	thioredoxin reductase 2, mitochondrial isoform X2
XP_011157958.1	232	1.11	chymotrypsin-1-like
XP_025987047.1	1290	1.08	neuroglian isoform X2
ADX94407.1	150	0.94	odorant-binding protein 11
XP_025988350.1	303	0.90	protein couch potato isoform X1
XP_011169374.1	135	0.86	ras-related protein Rab-8A isoform X1
XP_011156116.1	238	0.76	FK506-binding protein 2 isoform X1
XP_025996425.1	65	0.76	charged multivesicular body protein 4c, partial
XP_011171041.1	187	0.73	takeout-like
XP_025990129.1	322	0.73	fasciclin-1 isoform X4
XP_025987773.1	118	0.71	chymotrypsin-2-like, partial
XP_011158826.1	309	0.70	apoptosis-inducing factor 3 isoform X4
XP_011157318.1	46	0.68	60S ribosomal protein L34
XP_011170061.1	315	0.67	hexokinase type 2-like isoform X2
XP_011155994.1	41	0.66	thioredoxin domain-containing protein 17-like
XP_011162043.1	632	0.64	neprilysin-2 isoform X2
XP_011173656.1	73	0.62	40S ribosomal protein S24
XP_011162745.1	361	0.58	calpain-A isoform X6
XP_025988539.1	49	0.57	histidine triad nucleotide-binding protein 3
XP_011162536.1	93	0.55	dehydrodolichyl diphos. synthase subunit DHDDS-like
XP_025993927.1	104	0.54	ATP-dependent Clp protease proteolytic subunit
XP_025995049.1	125	0.54	ATP-dependent (S)-NAD(P)H-hydrate dehydratase
XP_011157133.1	128	0.52	glyoxylate reductase/hydroxypyruvate reductase isoform X2
XP_011159537.1	81	0.52	60S ribosomal protein L24
XP_025997729.1	53	0.52	cholinesterase, partial
XP_011158543.1	124	0.51	esterase-like
XP_025990759.1	131	0.51	cytochrome P450 4V2-like
XP_011165494.1	273	0.50	tropomyosin alpha-1 chain isoform X7
XP_011170800.1	75	0.49	AP-1 complex subunit sigma-2 isoform X1
XP_011175920.1	43	0.49	translocon-associated protein subunit delta
XP_011164789.1	36	0.48	immediate early response 3-interacting protein 1
XP_011164763.1	55	0.47	lysozyme
XP_011169037.1	75	0.47	signal recognition particle 9 kDa protein
XP_025988224.1	107	0.47	ER membrane protein complex subunit 3
XP_011160367.1	105	0.45	CKLF-like MARVEL TM domain-containing protein 4
XP_011166161.1	84	0.44	signal peptidase complex catalytic subunit SEC11A
XP_025993338.1	372	0.44	plasma membrane calcium-transporting ATPase 2 isoform X5
XP_011163259.1	44	0.43	HIG1 domain family member 1A, mitochondrial
XP_025986058.1	116	0.43	cytochrome P450 4C1-like
XP_025986150.1	124	0.43	RNA-binding protein Musashi homolog Rbp6 isoform X2
XP_025987060.1	116	0.42	retinol dehydrogenase 12-like
XP_011155614.1	76	0.41	inositol 2-dehydrogenase
XP_011170034.1	89	0.41	U6 snRNA-associated Sm-like protein LSm8

Footnotes: 1. Fifty most abundant worker antenna-specific proteins, based on emPAI. 2. Mascot score. 3. exponentially multiiplied protein abundance index, calculated with Mascot's algorithm. Chemosensory-related proteins are highlighted in yellow.

Table S1, continued. Male-specific antennal proteins[1]

Accession	Score[2]	emPAI[3]	Description
XP_011167760.1	224	7.11	myosin light chain alkali isoform X1
XP_025991010.1	468	7.11	RUS1 family protein homolog
XP_011160620.1	330	4.12	protein D2-like isoform X2, PE-binding protein
XP_025991263.1	267	3.14	venom allergen 2
XP_025992636.1	267	3.10	troponin I isoform X4
XP_011168081.1	197	3.01	mitochondrial import inner membrane translocase subunit Tim10
XP_011167550.1	2968	2.98	myosin heavy chain, muscle isoform X9
XP_011168538.1	916	2.52	thioredoxin reductase 1, mitochondrial isoform X3
AAC97370.1	92	2.24	venom allergen Sol i 4.02 precursor
XP_025988924.1	648	2.13	chitinase-like protein Idgf4 isoform X2
XP_011168088.1	46	2.00	28S ribosomal protein S36, mitochondrial isoform X1
XP_011171046.1	307	1.96	takeout-like
XP_011175494.1	172	1.85	tubulin-specific chaperone A
XP_011175241.1	332	1.70	serine protease inhibitor 3/4-like isoform X22
XP_011175227.1	400	1.67	serine protease inhibitor 3/4-like isoform X8
XP_011175946.1	2101	1.66	spectrin beta chain isoform X3
XP_011172292.1	218	1.57	synaptosomal-associated protein 25 isoform X2
XP_011169206.1	67	1.54	H/ACA ribonucleoprotein complex subunit 3
XP_025986856.1	356	1.52	acidic leucine-rich nuclear phosphoprotein 32 family member A
XP_011161615.1	163	1.50	mitochondrial intermembrane space import and assembly protein 40
XP_011169569.1	952	1.50	2-oxoglutarate dehydrogenase, mitochondrial isoform X2
XP_011161785.1	255	1.47	ubiquitin-conjugating enzyme E2 variant 2 isoform X2
XP_025990126.1	449	1.42	fasciclin-1 isoform X1
XP_011158824.1	451	1.38	apoptosis-inducing factor 3 isoform X1
XP_011161863.1	103	1.37	uncharacterized protein
XP_011169732.1	57	1.34	protein kish-A
XP_011170968.1	163	1.30	40S ribosomal protein S26
XP_011156923.1	118	1.22	ragulator complex protein LAMTOR2
XP_011164749.1	58	1.21	U1 small nuclear ribonucleoprotein C
XP_025985882.1	122	1.19	diamine acetyltransferase 2 isoform X2
XP_025993727.1	61	1.17	uncharacterized protein
XP_011159865.1	141	1.13	transcription factor A, mitochondrial isoform X2
XP_011171028.1	108	1.12	splicing factor 3B subunit 6
XP_011160131.1	105	1.10	myosin regulatory light chain sqh
XP_011164334.1	134	1.10	mesencephalic astrocyte-derived neurotrophic factor homolog
XP_011168534.1	121	1.10	fatty acid-binding protein homolog 5-like
XP_011163491.1	140	1.03	probable 28S ribosomal protein S16, mitochondrial
XP_011169534.1	67	1.03	barrier-to-autointegration factor
XP_011165226.1	112	1.01	ADP-ribosylation factor-like protein 8B-A
XP_011171574.1	58	1.00	peroxisomal acyl-coenzyme A oxidase 3-like, partial
XP_011165062.1	207	1.00	uncharacterized protein LOC105199602
XP_011165014.1	386	0.96	serine protease inhibitor 88Ea isoform X1
XP_011158539.1	80	0.93	synaptobrevin homolog YKT6
XP_011169660.1	232	0.92	UTP--glucose-1-phosphate uridylyltransferase isoform X2
XP_011161125.1	79	0.90	sleepless
XP_011167283.1	115	0.90	spermine synthase isoform X4
XP_025997646.1	84	0.90	28S ribosomal protein S18c, mitochondrial
XP_011161210.1	57	0.88	ragulator complex protein LAMTOR4 homolog
XP_011172073.1	103	0.87	prefoldin subunit 2
XP_011170023.1	121	0.85	transmembrane emp24 domain-containing protein bai isoform X2

Footnotes: 1. Fifty most abundant male antenna-specific proteins, based on emPAI. 2. Mascot score. 3. exponentially multiiplied protein abundance index, calculated with Mascot's algorithm. Chemosensory-related proteins are highlighted in yellow.

Table S2. Appendage-specific gene ontology terms

Worker Antenna and Male Antenna Only	
GO term	GO description
GO:0030042	actin filament depolymerization
GO:0042886	amide transport
GO:0006520	cellular amino acid metabolic process
GO:0022607	cellular component assembly
GO:0044085	cellular component biogenesis
GO:0016043	cellular component organization
GO:0071840	cellular component organization or biogenesis
GO:0034645	cellular macromolecule biosynthetic process
GO:0070727	cellular macromolecule localization
GO:0034641	cellular nitrogen compound metabolic process
GO:0034613	cellular protein localization
GO:0034622	cellular protein-containing complex assembly
GO:0051649	establishment of localization in cell
GO:0045184	establishment of protein localization
GO:0010467	gene expression
GO:0006886	intracellular protein transport
GO:0009059	macromolecule biosynthetic process
GO:0043170	macromolecule metabolic process
GO:0019941	modification-dependent protein catabolic process
GO:0016071	mRNA metabolic process
GO:0006397	mRNA processing
GO:0030837	negative regulation of actin filament polymerization
GO:0032272	negative regulation of protein polymerization
GO:0031333	negative regulation of protein-containing complex assembly
GO:1902904	negative regulation of supramolecular fiber organization
GO:0071705	nitrogen compound transport
GO:0006996	organelle organization
GO:0015833	peptide transport
GO:0051261	protein depolymerization
GO:0008104	protein localization
GO:0015031	protein transport
GO:0090066	regulation of anatomical structure size
GO:0032535	regulation of cellular component size
GO:0006511	ubiquitin-dependent protein catabolic process
GO:0016192	vesicle-mediated transport
**Worker Tibia Only**	
GO:0016052	carbohydrate catabolic process
GO:0006879	cellular iron ion homeostasis
GO:0043094	cellular metabolic compound salvage
GO:0055072	iron ion homeostasis

GO:0006740	NADPH regeneration
GO:0006098	pentose-phosphate shunt
GO:0043101	purine-containing compound salvage
**Male Antenna Only**	
GO:0030036	actin cytoskeleton organization
GO:0007015	actin filament organization
GO:0030041	actin filament polymerization
GO:0030029	actin filament-based process
GO:0008154	actin polymerization or depolymerization
GO:1901605	alpha-amino acid metabolic process
GO:0055082	cellular chemical homeostasis
GO:0022411	cellular component disassembly
GO:0044260	cellular macromolecule metabolic process
GO:0043624	cellular protein complex disassembly
GO:0006888	endoplasmic reticulum to Golgi vesicle-mediated transport
GO:0006188	IMP biosynthetic process
GO:0046040	IMP metabolic process
GO:0007005	mitochondrion organization
GO:0043632	modification-dependent macromolecule catabolic process
GO:0000398	mRNA splicing, via spliceosome
GO:0046112	nucleobase biosynthetic process
GO:0009123	nucleoside monophosphate metabolic process
GO:0017038	protein import
GO:0065003	protein-containing complex assembly
GO:0032984	protein-containing complex disassembly
GO:0043933	protein-containing complex subunit organization
GO:0032956	regulation of actin cytoskeleton organization
GO:0030832	regulation of actin filament length
GO:0110053	regulation of actin filament organization
GO:0030833	regulation of actin filament polymerization
GO:0032970	regulation of actin filament-based process
GO:0008064	regulation of actin polymerization or depolymerization
GO:0044087	regulation of cellular component biogenesis
GO:0051493	regulation of cytoskeleton organization
GO:0032271	regulation of protein polymerization
GO:0043254	regulation of protein-containing complex assembly
GO:1902903	regulation of supramolecular fiber organization
GO:0022618	ribonucleoprotein complex assembly
GO:0071826	ribonucleoprotein complex subunit organization
GO:0008380	RNA splicing
GO:0000375	RNA splicing, via transesterification reactions
GO:0000377	RNA splicing, via transest. reactions w/bulged A
GO:0009069	serine family amino acid metabolic process
GO:0000387	spliceosomal snRNP assembly

GO:0097435	supramolecular fiber organization
**Male Antenna and Worker Tibia Only**	
GO:1901137	carbohydrate derivative biosynthetic process
GO:0046916	cellular transition metal ion homeostasis
GO:0034220	ion transmembrane transport
GO:0006811	ion transport
GO:0009142	nucleoside triphosphate biosynthetic process
GO:0009141	nucleoside triphosphate metabolic process
GO:0006796	phosphate-containing compound metabolic process
GO:0006793	phosphorus metabolic process
GO:0009201	ribonucleoside triphosphate biosynthetic process
GO:0009199	ribonucleoside triphosphate metabolic process
**Worker Antenna and Worker Tibia Only**	
GO:0006508	proteolysis
GO:0006414	translational elongation
**Worker Antenna Only**	
GO:0043038	amino acid activation
GO:0006120	mitochondrial electron transport, NADH to CoQ
GO:0033365	protein localization to organelle
GO:0043039	tRNA aminoacylation
GO:0006418	tRNA aminoacylation for protein translation

Table S3. Venom protein isoforms detected on appendages

Quantities of venom proteins (exponentially multiplied protein abundance index, emPAI) detected in appendages compared with amounts previously measured in venom sacs.

	Venom allergen 2		Venom allergen 4		Ratios		
	major isoform[1]	minor isoform[2]	4.02[3]	4.01[4]	2 minor/ 2 major	4.02/ 2 major	4.01/ 2 major
Worker antenna	0. 8	0	0	0	0	0	0
Male antenna	3.2	1.8	1.0	0	0.55	0.31	0
Worker tibia	4.6	0	0	1.0	0	0	0.22
Worker venom sac[5]	3477	10	32	3.8	0.002	0.02	0.001

Footnotes: 1. emPAI values for accession number P35775.1. 2. emPAI values for accession number XP_025991263.1. 3. emPAI values for acession number AAC97370.1. 4. emPAI values for accession number P35777.2. 5. Data from Das et al. (Das et al. 2018).

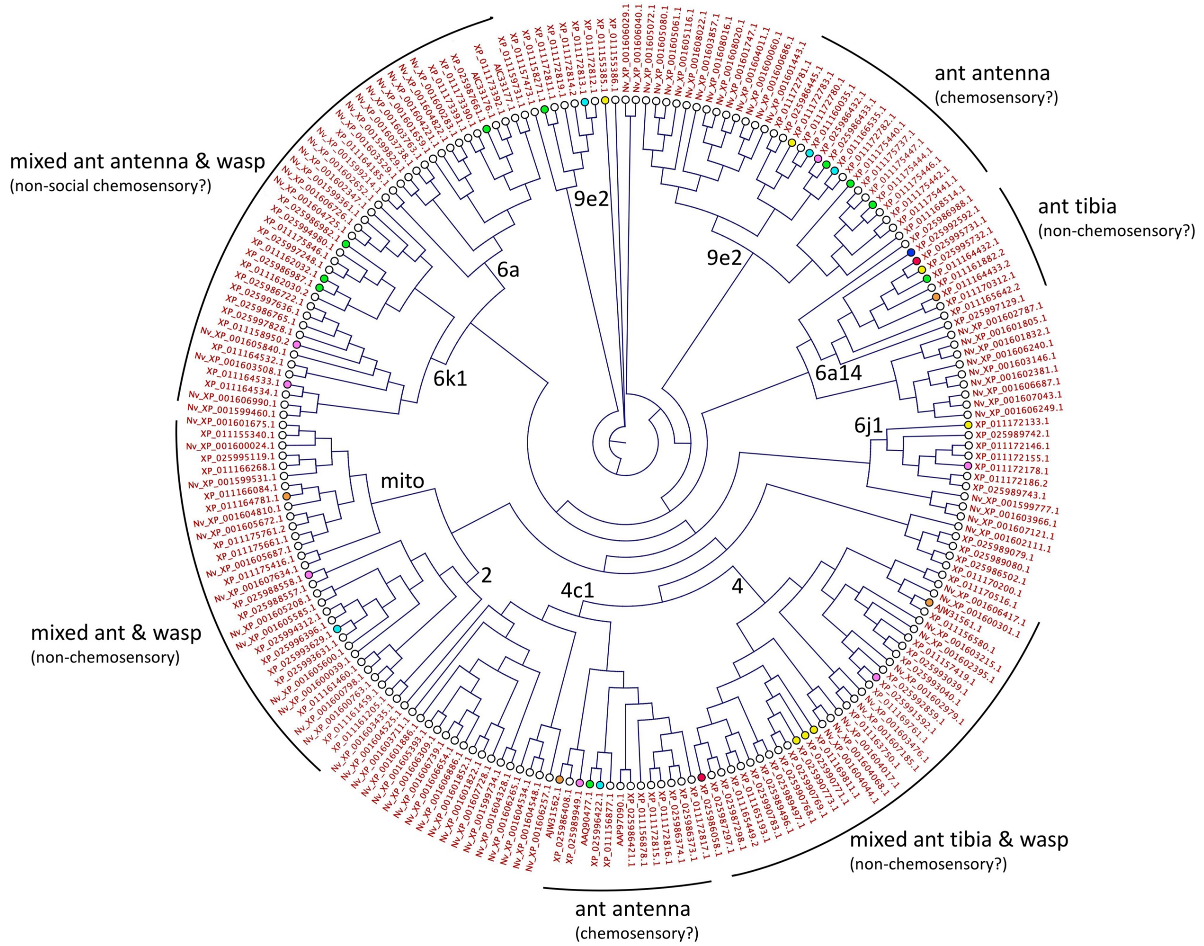

Supplementary Figure 1. Cladogram comparing *Nasonia vitripennis* and *Solenopsis invicta* cytochrome P450 sequences. *N. vitripennis* sequences from Oakeshott et al. (2010), with Nv_ preceeding the NCBI accession number (note: due to genome updating, some of these numbers have been changed in the current NCBI database). Light blue: male antenna; dark blue: male antenna and worker tibia; pink: worker antenna, red: worker antenna and worker tibia, green: both worker and male antenna; orange: worker tibia; yellow: male antenna, worker antenna, and worker tibia. Numbers on the branches indicate the cytochrome P450 type and, in some cases, the class. Curved lines mark clusters of *S. invicta* cytochrome P450s that are mostly antennal, mostly tibial, or combined.

References

Das, T, Alabi, I, Colley, M, Yan, F, Griffith, W, Bach, S, Weintraub, ST and Renthal, R. 2018. Major venom proteins of the fire ant *Solenopsis invicta*: insights into possible pheromone-binding function from mass spectrometric analysis. *Insect Mol Biol* **27**: 505-511.

Oakeshott, JG, Johnson, RM, Berenbaum, MR, Ranson, H, Cristino, AS and Claudianos, C. 2010. Metabolic enzymes associated with xenobiotic and chemosensory responses in *Nasonia vitripennis*. *Insect Mol Biol* **19** Suppl 1: 147-63.
